# Multimodal analysis of genome-wide methylation, copy number aberrations, and end motif signatures enhances detection of early-stage breast cancer

**DOI:** 10.3389/fonc.2023.1127086

**Published:** 2023-05-08

**Authors:** Thi Mong Quynh Pham, Thanh Hai Phan, Thanh Xuan Jasmine, Thuy Thi Thu Tran, Le Anh Khoa Huynh, Thi Loan Vo, Thi Huong Thoang Nai, Thuy Trang Tran, My Hoang Truong, Ngan Chau Tran, Van Thien Chi Nguyen, Trong Hieu Nguyen, Thi Hue Hanh Nguyen, Nguyen Duy Khang Le, Thanh Dat Nguyen, Duy Sinh Nguyen, Dinh Kiet Truong, Thi Thanh Thuy Do, Minh-Duy Phan, Hoa Giang, Hoai-Nghia Nguyen, Le Son Tran

**Affiliations:** ^1^ Medical Genetics Institute, Ho Chi Minh, Vietnam; ^2^ Research and Development Department Gene Solutions, Ho Chi Minh, Vietnam; ^3^ Ultrasound Department Medic Medical Center, Ho Chi Minh, Vietnam; ^4^ Department of Biostatistics, School of Medicine, Virginia Commonwealth University, Richmond, VA, United States; ^5^ Faculty of Medicine Nguyen Tat Thanh University, Ho Chi Minh, Vietnam

**Keywords:** breast cancer early detection, cfDNA (circulating cell-free DNA), ctDNA (circulating tumor DNA), end motif, whole genome methylation, SPOT-MAS (Screen for the Presence Of Tumor by DNA Methylation And Size), machine learning model, copy number aberration (CNA)

## Abstract

**Introduction:**

Breast cancer causes the most cancer-related death in women and is the costliest cancer in the US regarding medical service and prescription drug expenses. Breast cancer screening is recommended by health authorities in the US, but current screening efforts are often compromised by high false positive rates. Liquid biopsy based on circulating tumor DNA (ctDNA) has emerged as a potential approach to screen for cancer. However, the detection of breast cancer, particularly in early stages, is challenging due to the low amount of ctDNA and heterogeneity of molecular subtypes.

**Methods:**

Here, we employed a multimodal approach, namely Screen for the Presence of Tumor by DNA Methylation and Size (SPOT-MAS), to simultaneously analyze multiple signatures of cell free DNA (cfDNA) in plasma samples of 239 nonmetastatic breast cancer patients and 278 healthy subjects.

**Results:**

We identified distinct profiles of genome-wide methylation changes (GWM), copy number alterations (CNA), and 4-nucleotide oligomer (4-mer) end motifs (EM) in cfDNA of breast cancer patients. We further used all three signatures to construct a multi-featured machine learning model and showed that the combination model outperformed base models built from individual features, achieving an AUC of 0.91 (95% CI: 0.87-0.95), a sensitivity of 65% at 96% specificity.

**Discussion:**

Our findings showed that a multimodal liquid biopsy assay based on analysis of cfDNA methylation, CNA and EM could enhance the accuracy for the detection of early- stage breast cancer.

## Introduction

According to the Global Cancer Statistics 2020, breast cancer has the highest 5-year prevalence rate in both the U.S. and Vietnam, with about 640 people in the U.S. and 124 people in Vietnam having breast cancer per 100,000 people (1). Among all cancers in females, mortality caused by breast cancer ranked first, even surpassed lung cancer (1). National cost of medical services and prescription drugs for breast cancer in the U.S. in 2020 summed up to 28.6 billion dollars and is the costliest cancer in the U.S (2)..

Breast cancer is divided into five subtypes based on surface molecule expression: Luminal A, luminal B, human epidermal growth factor receptor 2 positive (HER2), luminal B-HER2, and triple negative breast cancer (TNBC) (3). Luminal A subtype is characterized by the presence of estrogen receptor (ER) or progesterone receptor (PR) or both, but with the absence of HER2, and low Ki-67 expression (4). Luminal B subtype is positive for ER or PR or both, also negative for HER2, but expresses high levels of Ki-67 (5). HER2 positive breast cancer is characterized by increased expression of HER2 and negative for ER and PR. HER2 tumor is also highly proliferative and often associated with poor prognosis (6). Luminal-HER2 (also known as luminal B-HER2) subtype is positive for both ER and HER2 and has worse prognosis than luminal A or B but better prognosis than HER2 positive (7, 8). TNBC refers to the subtype which stained negative for all three receptors ER, PR, and HER2. TNBC is aggressive and associated with adverse prognosis (9–11).

Breast cancer screening is recommended by the U.S. Preventive Services Task Force, the American Cancer Society, American College of Obstetricians and Gynecologists, American College of Radiology and more, although the age to start screening and frequency of screening vary between organizations (12–14). Mammography is currently the most common method for breast cancer screening and has significantly contributed to the reduction in breast cancer mortality rate (15). The major drawback of this method is the high false positive rate, especially in young women (<40 years old) who have dense breasts, resulting in overdiagnosis (16). In addition, exposure to ionizing radiation from repeated mammography screening may increase cancer risk (17). A second method commonly used for screening for breast cancer is Magnetic Resonance Imaging (MRI). Although MRI can avoid X-ray on the breast and generally gives higher sensitivity than mammography, it usually has lower specificity (18, 19). To address the clinical challenges, current research aims to develop alternative screening methods that are non-invasive and have the potential to improve sensitivity and specificity for detecting early-stage breast cancer.

Liquid biopsy refers to the sampling of body fluids such as plasma, urine, saliva, and cerebrospinal fluid (20). Liquid biopsy is not invasive, enables repeated sampling, and thus allows for screening and monitoring disease progression. Normal tissues and tumors release cell free DNA (cfDNA) to the bloodstream mainly through apoptosis and necrosis (21). The characterization of circulating tumor DNA (ctDNA) opens the door to many applications such as profiling tumor genetics, monitoring treatment response, and especially early cancer screening for asymptomatic individuals (22). Owing to the reflection of the genetic and epigenetic alterations in the tumor, ctDNA could serve as a specific and sensitive biomarker for improving early detection of breast cancer.

For early cancer screening, the goal is to detect the presence of ctDNA in the pool of cfDNA. Attempts to do so include mutation-based and non-mutation-based approaches (23). Our previous works have demonstrated the utility of massive parallel sequencing with unique molecular identifier tags for identification of tumor related mutations on cfDNA. We detected high concordance rates of mutation profiles between plasma cfDNA and matched tumor tissues in patients with NSCLC and colorectal cancer (24, 25). Compared to other cancer types, mutation-based breast cancer detection had lower sensitive, probably due to the inherent complexity of breast cancer with many subtypes and the low amount of ctDNA released by breast tumors in early disease stages (26, 27). Therefore, ultra-deep sequencing is required to increase the sensitivity of mutation detection, which is cost prohibitive for a cancer screening test.

As alternatives to mutation-based approaches, recent studies focus on other signatures of ctDNA such as methylation, fragment length (Flen), end motifs (EM), and copy number aberration (CNA). Methylation is an epigenetic marker of ctDNA with increasing utility in cancer detection, treatment response and relapse prediction (28–30). Liu et al. used data from The Cancer Genome Atlas to derive 9,223 consistently hypermethylated regions across 32 cancer types. Targeted bisulfite sequencing of cfDNA with those sites enabled correct identification of 91.7% patients with breast cancer (29). However, all breast patients recruited in this study were at advanced stages, thus the ability of this assay to detect early-stage breast cancer patients was not reported. Together, these studies demonstrated the feasibility of using ctDNA methylation for detecting breast cancer.

Multiple cfDNA fragmentomic features, including Flen and EM, have recently been explored to enhance the detection of early-stage cancers or low shedding ctDNA cancers (31–33). Liu et al. showed that cfDNA fragments carrying lung cancer associated mutations are in general shorter than non-cancer cfDNA fragments (31). Jiang et al. detected higher proportions of short cfDNA fragments (<150 bp) in plasma samples of hepatocellular carcinoma (HCC) patients compared to healthy individuals (32). Likewise, by performing whole genome sequencing (WGS), Cristiano et al. reported that fragment length profiles could be exploited to detect multiple cancer types including breast, colorectal, lung, ovarian, pancreatic, gastric and bile duct cancer (33). Recently, it has been shown that cancer and normal cells present different nucleosomal patterns and open chromatin regions, which determine the level of accessibility and cleavage sites for DNases (34, 35). Moreover, the activities of different DNases were also thought to differ between normal to malignant conditions (36, 37). Thus, such differences result in distinct landscapes of motif sequences at cfDNA fragment ends between cancer and normal cells, namely EM. Jiang et al. examined the 4-mer motifs at the 5’ end of cfDNA fragments in HCC patients and healthy individuals and found significant differences in frequencies of certain motifs between the two groups (38).

CNA in the cancer genome is associated with the initiation and progression of numerous cancers by altering transcriptional levels of both oncogenes and tumor suppressor genes (39). While examining CNA in HCC patients, Meng et al. found that the CNA profile of cfDNA in HCC patients correlated with the CNA profile of matched tumor tissue and was distinct from cfDNA CNA profile of healthy subjects (40). This study further demonstrated that CNAs detection could identify and quantify the fraction ctDNA in plasma cfDNA. The strategy of integrating different signatures of ctDNA to improve cancer prediction has been shown to be effective in many types of cancer. We previously showed that integration of genome-wide methylation (GWM), Flen, and targeted methylation of hypermethylated regions improved the accuracy for detecting colorectal cancer (41). Other research groups showed the integration of Flen and CNA (40, 42) or methylation and CNA (43) in prediction models improved HCC detection. Hence, we surmised that with breast cancer, a multimodal approach involving simultaneous analysis of multiple ctDNA signatures at genome-wide level may overcome the challenges of low abundance with ctDNA and heterogeneity with different molecular subtypes.

In this study, we employed the multimodal assay SPOT-MAS (Screening for the Presence Of Tumor by DNA Methylation And Size) to simultaneously profile methylomics, fragmentomics, DNA copy number and end motifs of cfDNA in a single workflow using targeted and shallow genome-wide sequencing. We extracted multiple cancer-specific signatures from 239 nonmetastatic breast cancer patients and 278 healthy subjects to train and validate this approach. To analyze such large multi-feature datasets of cfDNA, we exploited machine learning algorithms to combine them into a single screening test for breast cancer detection.

## Materials and methods

### Patient enrollment

A total of 239 breast cancer patients and 278 healthy subjects were recruited in this study. All breast cancer patients were confirmed to have breast cancer by abnormal mammograms and subsequent tissue biopsy confirmation of malignancy. Breast cancer stages were determined according to the American Joint Committee on Cancer and the International Union for Cancer Control (44). This study only recruited breast cancer patients with non-systemic-metastatic stages (Stage I-IIIA) in which cancer is localized to the breast and has not spread to other organs. We excluded patients who were diagnosed with metastatic stage IV breast cancer. All healthy subjects were confirmed to have no history of breast cancer at the time of enrollment. They were followed up at six months and one year after enrollment to ensure that they did not develop breast cancer. Clinical characteristics of each breast cancer and healthy participant were presented in **Table S1**.

Breast cancer patients and control were randomly divided into a discovery and a validation cohort. The discovery cohort comprised 167 breast cancer patients and 181 healthy controls, from which data were used for the selection of significant features and model construction. The validation cohort included 72 breast cancer patients and 97 healthy controls, from which data were used to validate model performance (**Table 1**). In the discovery cohort, the median age at diagnosis of breast cancer patients was 51 years, while healthy subjects had a comparable median age of 50 (**Table 1**). Cancer patients with stage I (19.2%) and stage II (40.7%) account for approximately 60% of all cancer patients, while 12% were diagnosed with stage IIIA. The remaining cancer patients (28.1%) did not have staging information due to missing records. Those patients conducted mammograms at the recruitment sites and agreed to participate in the study but later chose to undergo tissue biopsy at other clinics, resulting in missing histological records. Among cancer patients, 15% had luminal A, 23.4% had luminal B, 18% had luminal B-HER2, 13.8% had HER2, and 6.6% had TNBC (**Table 1**). Patients and healthy individuals in the validation cohort had comparable median age to those in the discovery cohort. The proportions of tumor stages and subtypes of the validation cohort were comparable to those of the discovery cohort (**Table 1**).

**Table 1:** Summary of participants’ clinical features in discovery and validation cohort.

Criteria		Discovery cohort (N=348)					Validation cohort (N=169)				
		Breast cancer (N = 167)		Healthy control (N = 181)		p-value (Breast cancer vs Healthy control)	Breast cancer (N = 72)		Healthy control (N = 97)		p-value (Breast cancer vs Healthy control)
		N	Percentage	N	Percentage		N	Percentage	N	Percentage	
Gender	Female	167	100.0%	181	100.0%		72	100.0%	97	100.0%	
	Male	0	0.0%	0	0.0%		0	0.0%	0	0.0%	
Age	Median	51		50		Mann-Whitney test p-value=0.22	51		50		Mann-Whitney test p-value=0.43
	Min	25		30			28		31		
	Max	80		75			78		77		
Stage	I, IA, IB	32	19.2%				12	16.7%			
	II, IIA, IIB	68	40.7%				32	44.4%			
	III, IIIA	20	12.0%				14	19.4%			
	NA	47	28.1%				14	19.4%			
Subtype	HER2	23	13.8%				16	22.2%			
	Luminal A	25	15.0%				8	11.1%			
	Luminal B	39	23.4%				25	34.7%			
	Luminal B-HER2	30	18.0%				7	9.7%			
	TNBC	11	6.6%				2	2.8%			
	NA	39	23.4%				14	19.4%			

This study also included 87 hepatocellular carcinoma (HCC) patients whose data were used to confirm previous findings and verify our analytical methods. Summary of clinical features for HCC group and clinical characteristics of each HCC patient were presented in **Table S2A and B**, respectively. In the HCC group, 23% were female, and 77% were male, ranging from 27 to 86 years old. The median age of HCC group was 58 years old. All HCC patients were naïve to treatment at the time of sample collection and confirmed to have non-metastatic tumors by imaging diagnosis (**Table S2B**).

All study subjects were recruited at two sites, including the Medic Medical Center and Medical Genetics Institute in Ho Chi Minh City, Vietnam, from May 2019 to November 2022. The methodologies conformed to the standards set by the Declaration of Helsinki. This study was approved by the Ethics Committee of the Medic Medical Center and Medical Genetics Institute, Ho Chi Minh City, Vietnam. This study was undertaken with the understanding and written consent of each subject. All breast cancer and HCC patients were naïve to treatment at the time of blood sample collection.

### Breast cancer subtype identification

Breast cancers were subdivided into 5 molecular subtypes including luminal A, luminal B, luminal B-HER2, HER2, and TNBC based on markers on immunohistochemical staining of tumor biopsy samples (45). Briefly, luminal A was characterized by the presence of ER and/or PR receptor, the absence of HER2 receptor, and less than 14% cells staining positive for Ki-67. Luminal B was identified by the presence of ER and/or PR receptor, absence of HER2 receptor, and Ki-67 signals in more than 14% cells. Luminal B - HER2 was identified with the presence of ER and/or PR receptor, and presence of HER2 receptor, with any level of Ki-67 expression. HER2 was characterized by the overexpression of HER2 receptor and the absence of both ER and PR receptors. TNBC was identified by the null expression of ER, PR, and HER2 receptors.

### Isolation of cfDNA

Each participant provided 10 ml of peripheral blood, which were collected in Cell-Free DNA BCT tube (Streck, USA) and subjected to two rounds of centrifugation (2,000 × g for 10 min and then 16,000 × g for 10 min) to separate plasma from blood cells. The plasma fraction was collected, aliquoted (1 ml per aliquot), and stored at -80°C. Cell free DNA extraction was performed using plasma aliquots with the MagMAX Cell-Free DNA Isolation kit (ThermoFisher, USA), following the manufacturer’s protocol. The acquired cfDNA was quantified using the QuantiFluor dsDNA system (Promega, USA).

### Bisulfite conversion and library preparation

Bisulfite conversion and purification of cell-free DNA samples were conducted using EZ DNA Methylation-Gold Kit (Zymo research, D5006, USA), following the manufacturer’s instructions. Subsequently, bisulfite-converted DNA samples were denatured to single stranded then processed with Adaptase™ technology adding adapters and unique dual indexes using xGen™ Methyl-Seq DNA Library Prep Kit (Integrated DNA Technologies, 10009824, USA). DNA concentrations were measured using the QuantiFlour dsDNA system (Promega, USA).

### Genome-wide hybridization and sequencing

After library construction, an equal amount of DNA of each library were pooled together and sequenced on DNBSEQ-G400 DNA sequencing system (MGI Tech, China) to generate sequencing data with 100-bp paired-end reads, at sequencing depth of ~20 million reads. All FASTQ files were examined by FastQC v 0.11.9 and MultiQC v 1.12 for quality.

### Genome-wide methylation analysis

For GWM analysis, we adopted the integrated bioinformatics pipeline Methy-pipe (46). Briefly, we performed the trimming step by Trimmomatic with default parameters to remove adapter sequence and low-quality bases at fragment ends. Sequence alignment was implemented by the built-in mapping algorithm and generated bsalign files. We segmented the whole genome into 2734 non-overlapping bins of 1Mb (1 million bases) long and calculated methylation ratios at these bins by built-in functions from Methy-pipe.


$$\text{Methylation ratio=}\frac{\text{methylated cytosine}\left(\text{C}\right)}{\text{methylated C}+\text{unmethylated C}}$$

Mean methylation ratio was calculated for each bin in both cancer and control groups and subsequently used to plot GWM density curves. Similarly, mean methylation ratio was calculated for bins of each breast cancer subtype then used to plot GWM density curves of subtypes.

### Fragment length analysis

For fragment length (Flen) distributions, we implemented an in-house python script to convert the bsalign files into BAM files. All read pairs from the BAM file with fragment length ranging from 100 bp to 250 bp were collected. In the range of 100 to 250 bp, there were 151 possible fragment lengths, starting from length of 100 bp, with 1 bp increment, up to 250 bp. With each length, the frequency of fragment (%) was calculated by getting the percentage of reads with each length to the total read count in the range of 100 to 250 bp. This calculation resulted in a feature vector of 151 dimensions. We plotted fragment length (bp) against frequency of fragment (%) to obtain a Flen distribution curve.

### Copy number aberration analysis

CNA analysis was performed using the R-package QDNAseq (47). The whole genome was again segmented into non-overlapping 1Mb bins. We excluded bins which felt into the low mappability and Duke blacklist regions (48). The number of reads mapped to each bin was calculated by the function “binReadCounts”, and GC-content correction was conducted by the function “estimateCorrection” and “correctBins”. Final CNA feature was derived by bin-wise normalizing and outlier smoothing with the function “normalizeBins” and “smoothOutlierBins”. This process resulted in a feature vector of a length of 2691 bins.

To plot a heatmap, CNA Z-score was calculated as:


$$\text{CNA Z}-\text{score}=\frac{\begin{array}{c} \text{CNA value in each bin of breast cancer }\\ -\;\text{CNA value in the corresponding bin of control} \end{array}}{\text{SD of the CNA value in corresponding bin of control}}$$

To find out which bins had significant DNA gain or loss in the cancer versus control group, CNA values in each bin of breast cancer samples were compared with corresponding values in control samples using Wilcoxon rank sum test. Bins with adjusted p-value (Benjamini-Hochberg correction) ≤ 0.05 were considered significant. Those with log_2_ fold change (cancer vs control) > 0 were categorized as significant increase. Those with log_2_ fold change (cancer vs control) < 0 were categorized as significant decrease.

### End motif analysis

During library preparation, Adaptase™ technology (Integrated DNA Technologies, USA) was used to ligate adapters to ssDNA fragments in a template-independent reaction. This step involved adding a random tail to the 5’ end of reverse reads. Although median length of the tail was 8 bp and thus allow trimming to obtain information good enough for other analysis, the random-length tails did not allow exact determination of the 5’ end of the reverse reads. Therefore, EM features were determined based on the genomic coordinate of the 5’ end of the forward reads. We determined the first 4-nucleotide sequence based on the human reference genome hg19. In 256 possible 4-nucleotide motifs, the frequency of each motif was calculated by dividing the number of reads carrying that motif by the total number of reads, generating an EM feature vector of a length of 256 for each sample.

### Construction of machine learning models

All samples in the discovery dataset with either cancer positive or negative labels were used for model training to classify if a sample is healthy or cancerous. For every feature type (GWM, CNA, and EM), three machine learning algorithms including Logistic regression (LR), Random Forest (RF) and Extreme Gradient Boosting (XGB) were examined. Hyperparameters were chosen for each learning algorithm by using the “GridSearchCV” function in the scikit-learn (v.1.0.2), with ‘CV’ parameter (cross-validation) set to 10. The best hyperparameters for each algorithm were found using function ‘best_params_’ implemented in GridSearchCV. After that, feature selection was performed in each algorithm as follow: (1) for LR, the “penalty” parameter with ‘l1’ (LASSO regression), ‘l2’ (Ridge regression) and ‘none’ (no penalty) was examined to select the setting with the best performance; (2) for RF and XGB, a “SelectFromModel” function with the ‘threshold’ set to 0.0001 was applied automatically to get all features that had importance weight. Subsequently, the three algorithms (LR, RF and XGB) with the best hyperparameters and selection of features were tested using k-fold cross validation approach on the training cohort with k-fold set to 10-fold, and ‘scoring’ parameter set to ‘roc_auc’. This split the data into 10 groups, in which 9 groups were fitted and the remaining group was evaluated, which resulted in 10 ‘roc_auc’ scores. The average of these scores was used to evaluate the prediction performance of each model. The model with the highest ‘roc_auc’ average score was chosen, which was XGB for all feature types. A final combination model was constructed by combining all probability scores from three features (GWM, CNA and EM) with LR as based algorithm, resulting in only one probability score for every sample. The model cut-off was set based on the threshold specificity of >90% or >95% to meet the requirement for early cancer detection assays. This combination model was applied to the independent validation dataset of 72 breast cancer patients and 97 healthy controls to evaluate its ability to classify breast cancer patients from controls.

### Statistical analysis

In this study, either the Wilcoxon Rank Sum test or t-test was used to find statistically significant different features between breast cancer and control. t-test was used whenever features followed normal distribution, otherwise Wilcoxon Rank Sum test was used. Kruskal-Wallis test was used to compare samples from two or more groups of independent observations. The Kolmogorov-Smirnov test was used to decide whether two randoms samples have the same statistical distribution. When applicable, p-values were corrected by the Benjamini-Hochberg correction for multiple comparisons (with a corrected p-value cutoff α ≤ 0.05). DeLong’s test was used to compare the differences between AUCs (49). All statistical analyses were carried out using R (4.1.0) with some common data analysis packages: ggplot2, pROC, and caret. 95% confident interval (95% CI) was presented in a bracket next to a value accordingly.

## Results

### The multimodal SPOT-MAS assay workflow in detecting early breast cancer

**Figure 1:**
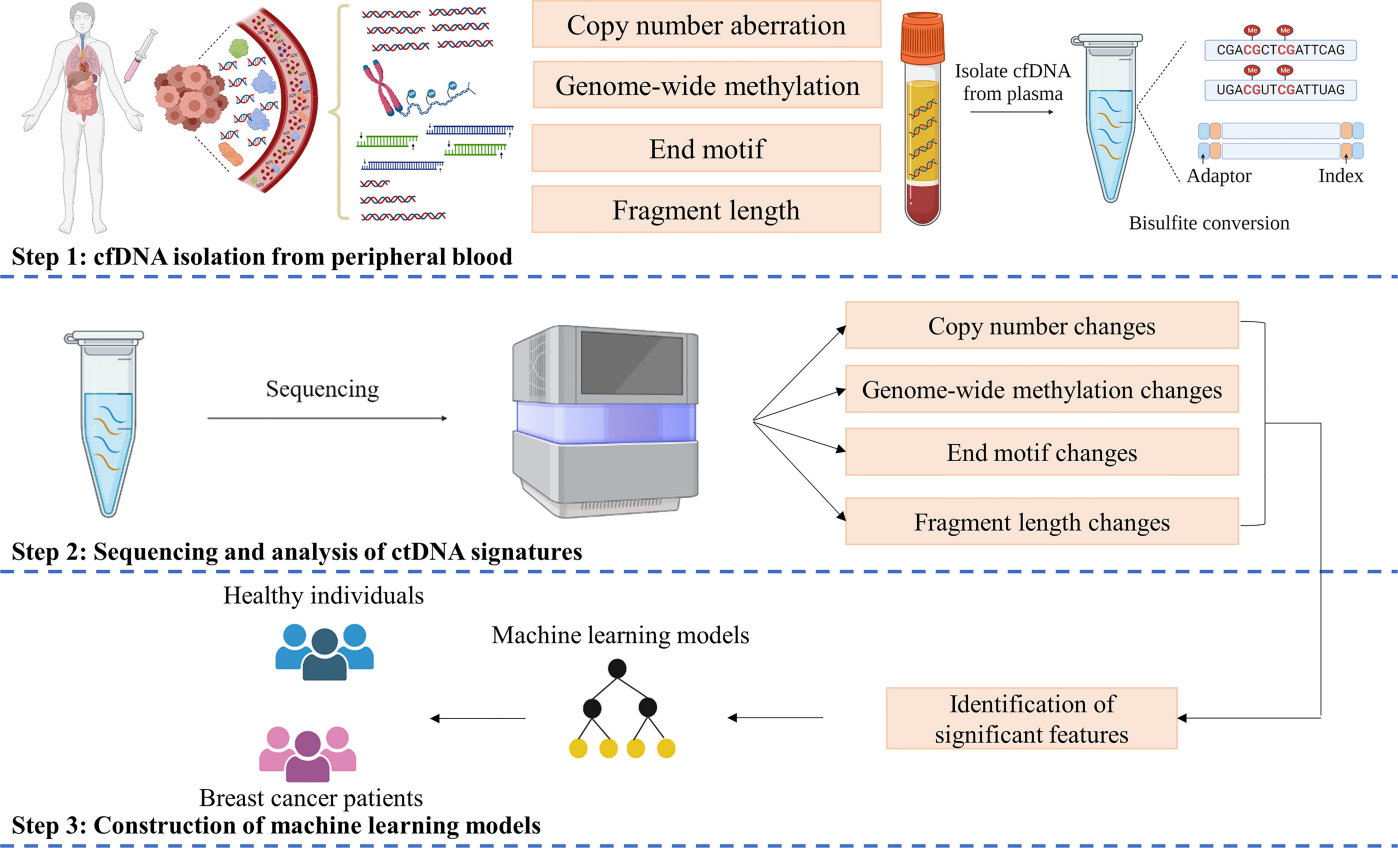
SPOT-MAS assay for detection of Breast cancer. In SPOT-MAS assay, cfDNA isolated from peripheral blood was subjected to bisulfite conversion, followed by library preparation including unique index and adapter ligation. DNA library was then subjected to massive parallel sequencing to examine four different features of cfDNA: copy number aberration (CNA), genome-wide methylation (GWM), end motif (EM) and fragment length profile (Flen). Machine learning models were subsequently constructed based on analysis of these features to distinguish breast cancer patients from healthy individuals.

We obtained higher concentrations of cfDNA from breast cancer patients than healthy subjects (**Figure S1A**). However, there was no significant difference among patients with different stages (**Figure S1A**) or subtypes (**Figure S1B**). We next employed a workflow named SPOT-MAS, which was described in our previous publication (41) to simultaneously analyze multiple signatures of ctDNA by performing low-cost, shallow whole-genome sequencing from a single library reaction (**Figure 1**). We achieved comparable sequencing depth coverages between breast cancer and healthy samples (0.58X for breast cancer and 0.57X for control, p=0.93, Wilcoxon test) (**Supplementary Figure S2A**). Apart from the GWM and Flen signatures reported in our previous study (41), in this study, we further characterized two other signatures including CNA and 4-mer EM and examined the possibility of incorporating all these features into a machine learning model to enhance the efficiency in detecting early-stage breast cancers.

### Fragment length of cfDNA was not different between breast cancer and healthy controls

It has been well established that cfDNA shed by cancer cells (ctDNA) tend to be shorter than cfDNA shed by other normal cells (33, 50). However, findings were still inconsistent across cancer types and different tumor stages (51). The aberrant size profile of DNA fragments in plasma of HCC patients compared to healthy individuals has been previously described in previous studies (32, 40, 52, 53). By using genome-wide sequencing, these studies consistently showed that the fragment size of HCC cfDNA was shorter than that of healthy individuals and short DNA fragments (< 150 bp) preferentially carried the tumor-associated genetic variations (52, 54). Therefore, we included cfDNA fragment length data of 87 HCC patients in this study to confirm previous findings and verify our analytical methods. Consistent with previous studies, we found that short DNA fragments (<150 bp) appeared more frequently in the plasma of HCC patients compared to healthy individuals (**Figure S2B**). By contrast, the distribution of fragment length was comparable between breast cancer and control, with median fragment length of 167.5 and 168 bp for breast cancer and control, respectively (p=0.99, Kolmogorov-Smirnov test, **Figure S2B**). Therefore, fragment length could not serve as a discriminative signature between early-stage breast cancer patients and healthy individuals.

### Plasma cfDNA from breast cancer patients displayed distinct patterns of DNA copy number aberration

**Figure 2:**
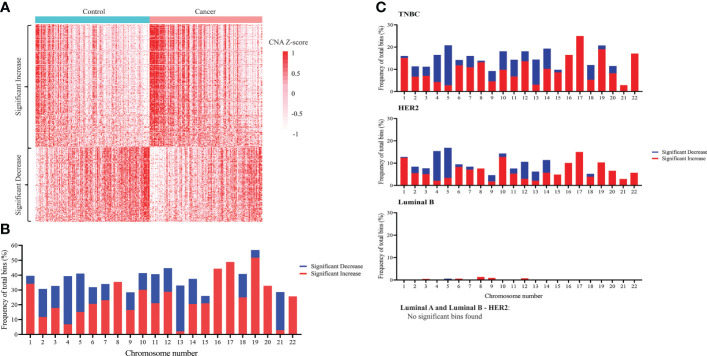
Genome-wide copy number changes in cfDNA of breast cancer patients and healthy controls. **(A)** Heatmap displaying CNA Z-scores (cancer vs control) of 616 significantly increased bins and 380 significantly decreased bins in 167 breast cancer patients and 181 healthy controls in the discovery cohort (t-test, Benjamini-Hochberg correction, p<0.05). **(B)** Frequency of bins with significant copy number decrease (blue) and copy number increase (red) across 22 chromosomes when comparing breast cancer with controls (Wilcoxon test, Benjamini-Hochberg correction, p<0.05). **(C)** Frequency of bins with significant copy number decrease (blue) and copy number increase (red) across 22 chromosomes when comparing each breast cancer subtype with controls. Significant copy number changes were found in TNBC, HER2, and luminal B subtypes (Wilcoxon test, Benjamini-Hochberg correction, p<0.05). No significant copy number changes were found in luminal A and luminal B – HER2 subtypes.

Since CNA is a hallmark of cancer (55–57), and CNA signal has been detected in ctDNA of different cancer types (53, 58, 59), we sought to compare CNA profiles between breast cancer patients and healthy subjects. Compared to cfDNA from healthy samples, breast cancer samples had 616 bins with significant increase and 380 bins with significant decrease in CNA, which could be used to differentiate them from healthy controls (**Figure 2A**). After mapping those significant bins to chromosomes, we observed significant copy number gains in all 22 chromosomes and copy number loss in 17/22 chromosomes except for chromosome 8, 16, 17, 20 and 22 in which CNA gain was solely detected (**Figure 2B**). When applying similar analysis on different subtypes of breast cancer, we found that breast cancer associated CNA pattern was reflected in TNBC and HER2 subtypes whereas it was less apparent in luminal B. Notably, no significant CNA changes were detected in the two remaining subtypes including luminal A and luminal B-HER2 subtypes (**Figure 2C**). This finding suggested that DNA copy number changes are heterogenous among different breast cancer subtypes with TNBC and HER2 subtypes showing CNA profiles distinct from healthy controls.

### Global hypermethylation was detected in cfDNA of breast cancer patients

**Figure 3:**
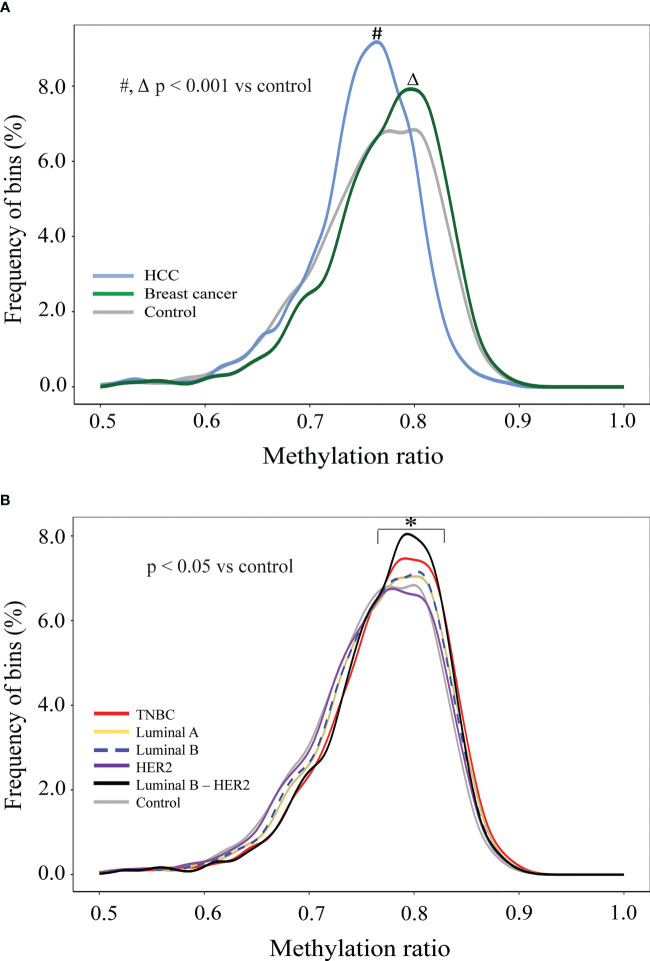
Genome-wide methylation changes in cfDNA of breast cancer patients in comparison to HCC patients and healthy individuals. **(A)** Density plots showing genome-wide methylation ratio distribution of HCC patients (light-blue curve), breast cancer patients (dark-green curve), and healthy controls (gray curve) (two-sample Kolmogorov-Smirnov test, p< 0.001). **(B)** Density plots showing genome-wide methylation ratio distribution of TNBC (red curve), luminal A (yellow curve), luminal B (blue dashed curve), HER2 (purple curve), luminal B – HER2 (black curve), and healthy controls (gray curve) (two-sample Kolmogorov-Smirnov test, p< 0.05).

To examine GWM, we calculated methylation ratio in each non-overlapping 1 Mb bin throughout the genome and plotted the frequency distribution of those values for healthy controls, breast cancer, and HCC (**Figure 3A**). The distribution of HCC methylation ratio shifted left-ward to the distribution of control (p< 0.001, Kolmogorov-Smirnov test), which indicated global hypomethylation, in agreement with previous studies (53, 60). On the other hand, by using the same analysis, we observed the curve of breast cancer shifted slightly right to that of control, which suggested that breast cancer samples displayed genome-wide hypermethylation (p< 0.001, Kolmogorov-Smirnov test), which was distinct from healthy individuals and HCC patients (**Figure 3A**). We next plotted the density curves of methylation ratio for each subtype against control. Among the five subtypes, TNBC and luminal B-HER2 displayed clear right skewness compared to control while the curve of HER2 overlapped with that of control, demonstrating the heterogeneity of global hypermethylation changes across different subtypes (**Figure 3B**). While hypomethylation at genome-wide scale in breast cancer has been reported in previous studies (61, 62), our analysis revealed higher proportions of hypermethylated bins in TNBC and Luminal B-HER2 as compared to healthy subjects.

### Breast cancer patients displayed plasma DNA 4-mer end motif profile distinct from healthy subjects

**Figure 4:**
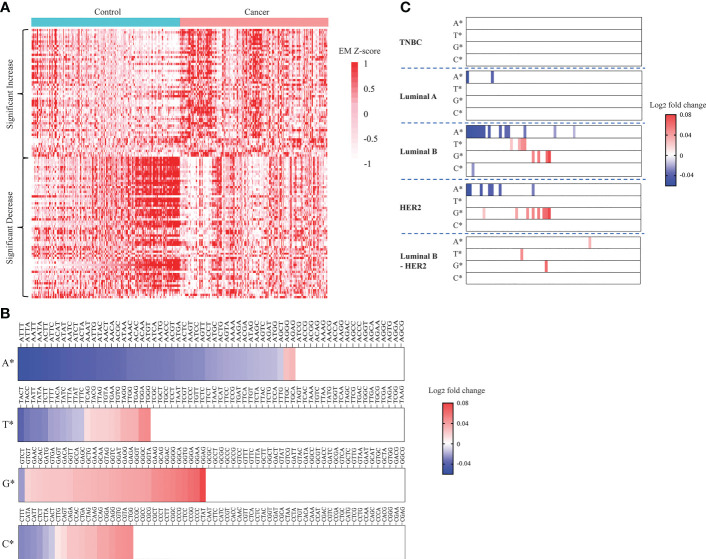
Landscapes of plasma cfDNA end motifs in breast cancer patients and healthy controls. **(A)** Heatmap displaying EM Z-scores (cancer vs control) of 56 EMs with significantly increased frequencies and 62 EMs with significantly decreased frequencies when comparing 167 breast cancer patients with 181 healthy controls in the discovery cohort (t-test, Benjamini-Hochberg correction, p<0.05). **(B, C)** Plots displaying log_2_ fold change (cancer vs control) in frequencies of the 256 EMs. EMs were grouped by the first nucleotides, being either A, T, G, or C. Log_2_ fold change in frequency was calculated for breast cancer vs controls **(B)** and each breast cancer subtype vs controls **(C)**, (t-test, Benjamini-Hochberg correction, p<0.05).

It was reported that cancer and healthy cfDNA differ in fragment size, nucleotide sequence at fragment end, jagged end, and genomic location of fragment endpoints (23). Of those fragmentomic signatures, cancer specific 4-mer EMs were previously reported to be different in HCC and healthy subject (38, 53). Here we examined whether breast cancer derived cfDNA fragments are enriched for any EMs. To do so, we compared the proportions of each of the 256 possible 4-mer motifs in cfDNA fragments of breast cancer and healthy samples. We found that 56 motifs appearing more frequently in breast cancer samples compared to healthy controls while 62 motifs appearing more frequently in healthy controls compared to breast cancer (**Figure 4A**). Interestingly, most EMs with increased frequency in breast cancer began with guanine (G). Of those, GGAG, GGAA, GGGA, GGTG and GGCA were identified as the five most significant increased motifs in breast cancer (**Figure 4B**; **Figure S3A**). On the other hand, EMs with significantly reduced frequency in breast cancer tended to begin with adenine (A). Of those, ATTT, ATTA, AATT, AATA and ACTT were identified as the five most significantly decreased motifs in breast cancer (**Figure 4B**; **Figure S3B**). Those significant EM signatures were clearly detected in luminal B and HER2 while luminal A and luminal B-HER2 had fewer significant changes in EM. By contrast, we did not find any significant EM change in TNBC samples (**Figure 4C**). These findings suggested that preferred end motifs of cfDNA fragments could serve as potential signatures to differentiate breast cancer, particularly those with luminal B and HER2 subtypes, from healthy individuals.

### A multimodal analysis combining genome-wide methylation, copy number aberration, and end motifs enhances the accuracy for breast cancer detection

**Figure 5:**
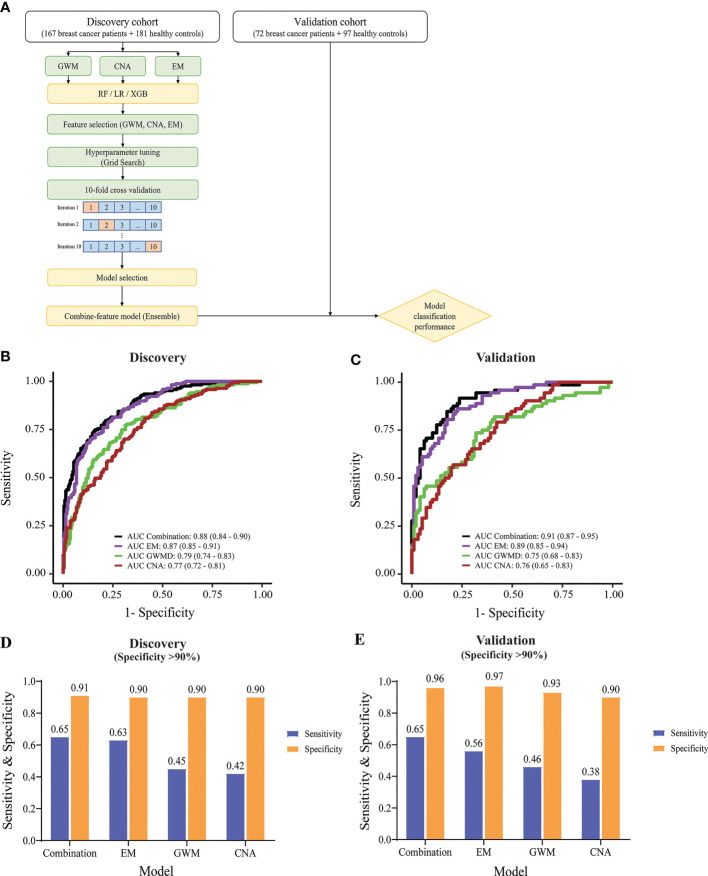
Construction and evaluation of machine learning classifiers to detect breast cancer. **(A)** Model construction workflow. **(B)** & **(C)** ROC curves showing the performance of the combination model and individual-feature models in the discovery cohort **(B)** and validation cohort **(C)**. **(D, E)** Bar graphs displaying sensitivity and specificity values, when specificity was set as at least 90%, for the combination and individual-feature models in the discovery cohort **(D)** and validation cohort **(E)**.

The identification of significant signatures including CNA, GWM and EM by genome-wide sequencing prompted us to build a classification model to distinguish breast cancer patients from healthy people. We employed three different machine learning algorithms including RF, LR, and XGB to build classification models using data from CNA, GWM and EM as inputs. Model construction workflow was summarized in **Figure 5A**. Briefly, we performed feature selection, hyperparameter tuning, 10-fold cross validation, and chose the best performing model with the highest area under the curve (AUC) value. Subsequently, we employed an ensemble method stacking probabilities of base models to build a combination model. Performance of the combination model and different base models were externally validated using data from the validation cohort.

XGB performed better than RF and LR, generating higher AUC values in all tested cfDNA features in receiver operating characteristic (ROC) plots (**Figure S4A**). XGB achieved AUC of 0.79 (95% CI: 0.74-0.83), 0.77 (95% CI: 0.72-0.81), and 0.87 (95% CI: 0.84-0.9) for GWM, CNA, and EM respectively (**Figure 5B**). Hence, we selected XGB to construct classification models.

In the discovery cohort, EM achieved the highest performance among three significant ctDNA features (**Figure 5B**). The combination and EM base model achieved comparable performance with AUC of 0.88 (95% CI: 0.84-0.9) and 0.87 (95% CI: 0.85-0.91) respectively. They performed significantly better than GWM (AUC of 0.79, 95% CI: 0.74-0.83) and CNA (AUC of 0.77, 95% CI: 0.72-0.81) base models (p<0.01, DeLong’s test) (**Figure 5B**). In the validation cohort, the combination model and EM base model also achieved consistent performance, with AUC of 0.91 (95% CI: 0.87-0.95) and 0.89 (95% CI: 0.85-0.94) respectively, which were better than GWM and CNA base models in the validation cohort (**Figure 5C**).

Furthermore, we observed that combining EM with other ctDNA features in the combination model could further enhance the sensitivity of detection at comparable specificity. The model cutoff value was set to achieve a specificity of at least 90% to minimize overdiagnosis and reduce false positive rates. In the discovery cohort with a specificity of at least 90%, the combination model and EM base model had much better sensitivity than GWM and CNA model (**Figure 5D**). Notably, the combination model showed better sensitivity than the EM base model (65% sensitivity at 91% specificity versus 63% sensitivity at 90% specificity) (**Figure 5D**). Consistently, in the validation cohort, with the same cutoff value, the combination model also achieved higher sensitivity than other models (65% sensitivity at 96% specificity versus the second best 56% sensitivity at 97% specificity of EM base model) (**Figure 5E**). When specificity was set as at least 95%, the combination model also achieved the highest sensitivity over ME, GWM, and CNA in both discovery and validation cohort (**Figure S4B**).

Together, machine learning using XGB and the multimodal input of three features of cfDNA yielded the best performing classification model for distinguishing breast cancer from control.

### The multimodal assay enabled effective detection of breast cancer at early stages and with heterogenous molecular subtypes

**Figure 6:**
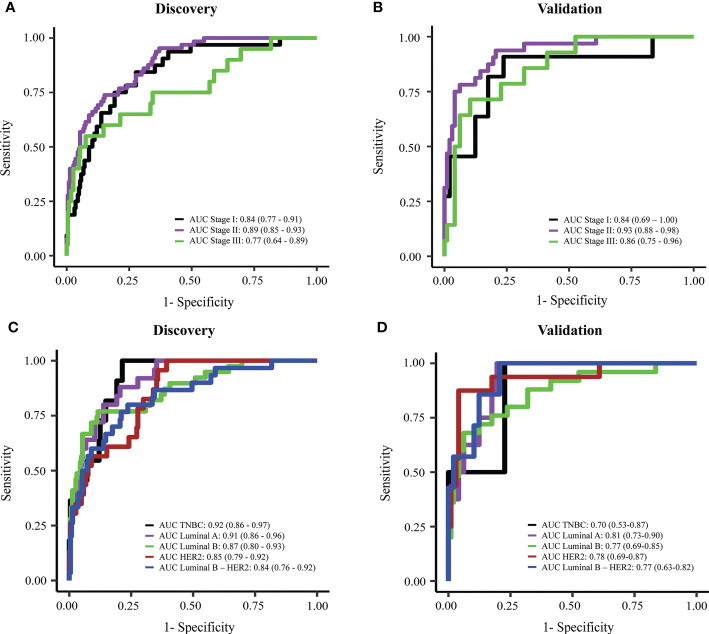
Performance of the combination model in breast cancer detection at different stages or with different subtypes. **(A, B)** ROC curves showing the performance of the combination model for breast cancer stage I to III in discovery cohort **(A)** and validation cohort **(B)**. **(C, D)** ROC curves showing the performance of the combination model on different breast cancer subtypes in discovery cohort **(C)** and validation cohort **(D)**.

Early breast cancer refers to breast cancer that has not spread beyond the breast or the axillary lymph nodes; this includes stage I, stage IIA, stage IIB, and stage IIIA breast cancers. The detection of early-stage breast cancer remains a major challenge in the field due to the low abundance of ctDNA in the blood (26, 27). Hence, we tested the efficacy of the combination model in detecting breast cancer at different stages. In the discovery cohorts, our combination model showed good performance in detecting stage I and II tumors, with an AUC of 0.84 (95% CI: 0.77-0.91) and 0.89 (95% CI: 0.85-0.93) for stage I and stage II respectively (**Figure 6A**). Model performance on stage I and stage II were slightly better than on stage III where AUC was 0.77 (95% CI: 0.64-0.89) in the discovery cohort (**Figure 6A**). In the validation cohort, the combination model achieved consistent performance on breast cancer patients with different stages, with AUCs for stage I, II, and III of 0.84 (95% CI: 0.69-1), 0.93 (95% CI: 0.88-0.98), and 0.86 (95% CI: 0.75-0.96) respectively (**Figure 6B**).

Breast cancer is known as a highly heterologous disease which comprises multiple subtypes manifested by distinct molecular alterations (4–8). Hence, we assessed whether our models preferred to detect a particular subtype. We did not observe any significant differences between AUC values for detecting five different subtype groups both in the discovery and validation cohort (**Figures 6C, D**). Notably, compared to individual-feature models, the combination model enabled better efficiencies in detecting overall breast cancer, and luminal B, HER2 and luminal B-HER2 subtypes (**Table 2**). This finding further confirmed that the combination of multiple ctDNA specific signatures could overcome the heterogeneity of breast tumors, thus enhancing the overall efficiency of breast cancer detection.

**Table 2:** Detection rate of overall breast cancer or each subtype.

Model	Discovery						Validation					
	All n=167	TNBC n=11	Luminal A n=25	Luminal B n=39	HER2 n=23	Luminal B - HER2 n=30	All n=72	TNBC n=2	Luminal A n=8	Luminal B n=25	HER2 n=16	Luminal B - HER2 n=7
**Combination**	65%	55%	64%	72%	57%	60%	65%	50%	50%	60%	88%	57%
**ME**	63%	55%	68%	69%	52%	57%	56%	50%	38%	48%	81%	71%
**GWM**	45%	45%	36%	44%	39%	33%	46%	50%	50%	48%	25%	14%
**CNA**	42%	73%	36%	23%	39%	57%	38%	50%	50%	40%	19%	14%

## Discussion

This study applied a multimodal approach, known as SPOT-MAS, to integrate three significant signatures of ctDNA including CNA, GWM, and EM to construct a multi-featured combination machine learning model to classify nonmetastatic breast cancer patients from healthy subjects. The approach enables increased breadth of ctDNA analysis at shallow sequencing depth to overcome the limitation of low amount of ctDNA fragments in plasma samples as well as molecular heterogeneity of breast cancers.

Our analysis of CNA showed significant changes across 22 chromosomes of breast cancer group compared to control. We also observed that different breast cancer subtypes had varied levels of CNA, in which TNBC and HER2 had remarkably higher numbers of bins with copy number changes than other subtypes. Our finding was consistent with previous studies showing that TNBC and HER2 subtype displayed genomic instability manifested by pronounced DNA copy gains and losses across the entire genome, dominantly gain in chromosome 1, 8, 17 and dominantly loss in chromosome 4, 5, and 13 (63, 64). Such CNA profiles are associated with the high rate of relapse and the poor prognosis of these two subtypes (63, 64). Interestingly, we did not detect bins with significant copy changes for luminal A and luminal B-HER2 subtypes, suggesting that these two subtypes might have lower levels of genomic instability, which might be linked to the less aggressive nature of those subtypes compared to TNBC or HER2 (10).

HCC patients, who were previously shown to display genome-wide signatures such as fragment length and methylation markedly distinct from healthy subjects (53, 60, 65) were recruited to confirm previous findings and validate our analysis on those features.

It has been reported that cancer patients display hypomethylated patterns at genome-wide levels compared to healthy subjects (66, 67), which was consistently observed in our analysis by comparing GWM between HCC patients and healthy controls. Interestingly, we observed an opposite trend for breast cancer which displayed hypermethylation across the entire genome compared to control. Thus, our finding suggested that global hypomethylation pattern might depend on the cancer type, which agrees with a previous study (68, 69). Paradoxically, several previous studies reported global hypomethylation in plasma cell free DNA of breast cancer patients (70–72). The discrepancy could be attributed to differences in tumor stages or mutation status of cancer patients between our cohorts and others. Using paired-end whole-genome bisulfite sequencing (WGBS), Legendre et al. (66) detected global hypomethylation in cfDNA of breast cancer with metastatic tumors while our study analyzed nonmetastatic breast cancer. In support to this notion, a previous study showed that the methylation pattern of breast cancer DNA changes during breast tumor progression, with pronounced global methylation in the metastatic stage (73, 74) while early-stage tumors tend to display hypermethylation. Furthermore, it is known that DNA methylation is mediated by DNA methyltransferases (DNMTs) and that mutations in genes encoding DNMTs could alter DNA hypermethylation or hypomethylation profiles of breast tumors (74). Thus, further studies are required to profile the mutational landscape of *DNMT* genes and assess whether such mutations, if detected, are associated with the global hypermethylation patterns of breast cancer patients in our cohort, particularly those with TNBC and luminal B-HER2 subtypes. Not only at genome-wide scale, site-specific methylation changes between breast cancer patients and healthy controls have been reported in numerous studies. Those regions are responsible for major changes in regulating expression of cancer associated genes by impacting the binding of different transcription factors to regulatory sites and chromatin structure (75). Therefore, we established a panel of 16 regions based on previous publications and those regions were mapped to regulatory regions including promoters or enhancers of tumor suppressor genes involving cancer progression (**Table S3**). Of those 16 tested regions, 4 (SOX17_1, RASSF1A, OTX2_1 and OTX2 _2) were confirmed to be significantly hypermethylated in breast patients as compared to healthy subjects (p<0.005, **Figure S5**). Thus, our ongoing study will examine whether these hypermethylation DMRs could be combined with other genome-wide signatures of cfDNA to improve the accuracy of our assay for detection of breast cancer patients.

In our previous study, we employed SPOT-MAS workflow to compare the fragment length profiles of cfDNA between CRC patients and healthy individuals (41). In this study, we performed a similar analysis on HCC and breast cancer patients. Consistent with CRC patients, we observed higher frequencies of short fragments (<150 bp) in cfDNA of HCC patients compared to healthy individuals. However, we did not detect any differences in the fragment length distribution between breast cancer and healthy controls. Thus, it is possible that the low abundance of breast cancer ctDNA precludes the detection of cancer associated fragment length patterns which could be readily detected in other types of cancer shedding higher amounts of ctDNA into the bloodstream.

In this study, we identified significant EMs enriched in breast cancer ctDNA, further confirming that the fragmentation process of breast cancer ctDNA is a nonrandom process (76). We showed that the feature of EM achieved higher performance than other ctDNA signatures including CNA and GWM in discriminating breast cancer from healthy controls. Of all significant motifs, the top five most significant frequency increase and decrease were different from the represented motifs for HCC (57). Thus, our results suggested that EMs could also serve as novel cancer type specific signatures. Comparing different breast cancer subtypes, we found that significant EMs were not detected for all five breast cancer subtypes. In fact, luminal B and HER2 had the highest numbers of significant EMs among all five subtypes, while no significant motif was detected with significant change in TNBC, further highlighting the heterogeneity of breast cancer.

Despite having higher performance compared to other signatures, we found that combining EM with other ctDNA signatures could further improve the sensitivity for detecting breast cancer samples in the validation cohort. In support of this notion, we found that the combination model had higher detection rate for luminal B and HER2 subtypes compared to individual-feature modes in both discovery and validation cohort. These findings highlighted that the multimodal analysis of multiple ctDNA signatures could overcome the molecular heterogeneity of breast cancer, thus enhancing the detection sensitivity. We also compared the performance of our assays with other available ctDNA-based tests using single features of cfDNA (**Table S4**). Our test had comparable specificity (96% versus >98.0%, **Table S4**) to the other tests, while it demonstrated higher sensitivity (65% versus<60%, **Table S4**). Importantly, our test used a shallow depth sequencing approach with a lower depth coverage of 0.58X compared to other assays, resulting in economically feasible for population-wide screening (**Table S4**). Despite promises, it is important to note that current liquid biopsy tests should only be used as a complementary approach to the available diagnostic tests to increase rates of cancer detection, especially in the early stage. To be successfully applied to the clinical programs as a screening test, SPOT-MAS would need further work to improve its performance, focusing on high sensitivity and specificity. Moreover, prospective studies are required to validate the performance and cost-effectiveness of the test in the clinical setting.

There are several limitations to this study. First, this was a retrospective study, with small sample sizes for breast cancer and control groups as well as small number of samples for each breast cancer subtype in both discovery and validation cohorts. Thus, our current study could be considered as exploratory analyses and future studies with a larger prospective cohort are required for robust validation of clinical performance of our assay. Second, this study did not include benign breast tumor, thus the ability of the model to distinguish between benign breast lesions and breast cancer was not tested. Although patients with early-stage cancer (stage I and II) account for the majority (>60%), the remaining patients were either diagnosed with nonmetastatic tumor (stage IIIA) or had unknown staging status. However, we did not observe significant differences in the efficiencies of our assay in detecting patients with stage I/II and those with stage IIIA. Another limitation of our study is the reduction of the performance of different cancer subtypes in the validation cohort. This is attributed to the limited sample size available for each subtype in this cohort. To address the limitation, future studies should consider incorporating a more diverse range of samples representing different cancer subtypes. This would enable a comprehensive and robust evaluation of the performance of the SPOT-MAS test for detecting and classifying various cancer subtypes.

In summary, this study employed the multimodal approach of SPOT-MAS assay to profile multiple ctDNA signatures across the entire genome and incorporate those features in a machine learning model to develop a blood-based assay for breast cancer screening. We demonstrated that combining EM, CNA and GWM features could achieve high accuracy for detecting nonmetastatic breast cancer with different molecular subtypes.

## Data availability statement

The datasets generated for this study can be found in the NCBI BioProject, accession number: PRJNA929650. Link: https://www.ncbi.nlm.nih.gov/bioproject/PRJNA929650/.

## Ethics statement

The studies involving human participants were reviewed and approved by Ethics Committees of the Medic Medical Center, Ho Chi Minh City, Vietnam and Medical Genetics Institute, Ho Chi Minh City, Vietnam. The patients/participants provided their written informed consent to participate in this study.

## Author contributions

TMQP and TTTT did formal analysis and original draft preparation. THP did conceptualization. TXJ, TLV, THTN, TTT, MHT and NCT did methodology, formal analysis, patient recruitment and screening. LAKH, THN and TDN did formal analysis and software. TTTT, VTCN, THHN and NDKL did methodology and formal analysis. DSN, TTTD, MDP, HG, HNN and LST did supervision. HNN did funding acquisition. LST did revision, and edition of the manuscript. All authors contributed to the article and approved the submitted version.

## References

[1] Sung H Ferlay J Siegel RL Laversanne M Soerjomataram I Jemal A. Global cancer statistics 2020: GLOBOCAN estimates of incidence and mortality worldwide for 36 cancers in 185 countries. Cancer J Clin (2021) 71(3):209–49. 10.3322/caac.21660 33538338

[2] Mariotto AB Enewold L Zhao J Zeruto CA Yabroff KR. Medical care costs associated with cancer survivorship in the united states. Cancer Epidemiol Biomarkers Prev (2020) 29(7):1304–12. 10.1158/1055-9965.EPI-19-1534 PMC9514601 32522832

[3] Goldhirsch A Wood WC Coates AS Gelber RD Thürlimann B Senn HJ. Strategies for subtypes–dealing with the diversity of breast cancer: highlights of the st. gallen international expert consensus on the primary therapy of early breast cancer 2011. Ann Oncol (2011) 22(8):1736–47. 10.1093/annonc/mdr304 PMC3144634 21709140

[4] Gao JJ Swain SM. Luminal a breast cancer and molecular assays: a review. Oncologist (2018) 23(5):556–65. 10.1634/theoncologist.2017-0535 PMC5947456 29472313

[5] Ades F Zardavas D Bozovic-Spasojevic I Pugliano L Fumagalli D de Azambuja E. Luminal b breast cancer: molecular characterization, clinical management, and future perspectives. J Clin Oncol (2014) 32(25):2794–803. 10.1200/JCO.2013.54.1870 25049332

[6] Schlam I Swain SM. HER2-positive breast cancer and tyrosine kinase inhibitors: the time is now. NPJ Breast Cancer (2021) 7(1):56. 34016991 10.1038/s41523-021-00265-1 PMC8137941

[7] Cheang MC Chia SK Voduc D Gao D Leung S Snider J. Ki67 index, HER2 status, and prognosis of patients with luminal b breast cancer. J Natl Cancer Inst (2009) 101(10):736–50. 10.1093/jnci/djp082 PMC2684553 19436038

[8] Piccart M Lohrisch C Di Leo A Larsimont D. The predictive value of HER2 in breast cancer. Oncology (2001) 61 Suppl 2:73–82. 11694791 10.1159/000055405

[9] Perou CM Sørlie T Eisen MB van de Rijn M Jeffrey SS Rees CA. Molecular portraits of human breast tumours. Nature (2000) 406(6797):747–52. 10.1038/35021093 10963602

[10] Yersal O Barutca S. Biological subtypes of breast cancer: prognostic and therapeutic implications. World J Clin Oncol (2014) 5(3):412–24. 10.5306/wjco.v5.i3.412 PMC4127612 25114856

[11] Foulkes WD Smith IE Reis-Filho JS. Triple-negative breast cancer. N Engl J Med (2010) 363(20):1938–48. 10.1056/NEJMra1001389 21067385

[12] Siu AL. Screening for breast cancer: U.S. preventive services task force recommendation statement. Ann Intern Med (2016) 164(4):279–96. 10.7326/M15-2886 26757170

[13] Oeffinger KC Fontham ET Etzioni R Herzig A Michaelson JS Shih YC. Breast cancer screening for women at average risk: 2015 guideline update from the American cancer society. Jama (2015) 314(15):1599–614. 10.1001/jama.2015.12783 PMC4831582 26501536

[14] Monticciolo DL Newell MS Hendrick RE Helvie MA Moy L Monsees B. Breast cancer screening for average-risk women: recommendations from the ACR commission on breast imaging. J Am Coll Radiol (2017) 14(9):1137–43. 10.1016/j.jacr.2017.06.001 28648873

[15] Løberg M Lousdal ML Bretthauer M Kalager M. Benefits and harms of mammography screening. Breast Cancer Res (2015) 17(1):63. 25928287 10.1186/s13058-015-0525-z PMC4415291

[16] Yankaskas BC Haneuse S Kapp JM Kerlikowske K Geller B Buist DS. Performance of first mammography examination in women younger than 40 years. J Natl Cancer Inst (2010) 102(10):692–701. 20439838 10.1093/jnci/djq090 PMC2902813

[17] National Research Council Board on Radiation Effects R. Health risks from exposure to low levels of ionizing radiation: BEIR VII, phase I, letter report (1998). Washington (DC: National Academies Press (US) Copyright © National Academy of Sciences (1998). 25077203

[18] Gundry KR. The application of breast MRI in staging and screening for breast cancer. Oncol (Williston Park) (2005) 19(2):159–69. 15770888

[19] Berg WA Zhang Z Lehrer D Jong RA Pisano ED Barr RG. Detection of breast cancer with addition of annual screening ultrasound or a single screening MRI to mammography in women with elevated breast cancer risk. JAMA (2012) 307(13):1394–404. 10.1001/jama.2012.388 PMC3891886 22474203

[20] Escudero L Martínez-Ricarte F Seoane J. ctDNA-based liquid biopsy of cerebrospinal fluid in brain cancer. Cancers (Basel) (2021) 13(9):1989. 33919036 10.3390/cancers13091989 PMC8122255

[21] Gormally E Caboux E Vineis P Hainaut P. Circulating free DNA in plasma or serum as biomarker of carcinogenesis: practical aspects and biological significance. Mutat Res (2007) 635(2-3):105–17. 10.1016/j.mrrev.2006.11.002 17257890

[22] Alix-Panabières C Pantel K. Clinical applications of circulating tumor cells and circulating tumor DNA as liquid biopsy. Cancer Discovery (2016) 6(5):479–91. 10.1158/2159-8290.CD-15-1483 26969689

[23] Lo YMD Han DSC Jiang P Chiu RWK. Epigenetics, fragmentomics, and topology of cell-free DNA in liquid biopsies. Science (2021) 372(6538):eaaw3616. 33833097 10.1126/science.aaw3616

[24] Tran LS Pham HT Tran VU Tran TT Dang AH Le DT. Ultra-deep massively parallel sequencing with unique molecular identifier tagging achieves comparable performance to droplet digital PCR for detection and quantification of circulating tumor DNA from lung cancer patients. PloS One (2019) 14(12):e0226193. 31841547 10.1371/journal.pone.0226193 PMC6913927

[25] Nguyen HT Tran DH Ngo QD Pham HT Tran TT Tran VU. Evaluation of a liquid biopsy protocol using ultra-deep massive parallel sequencing for detecting and quantifying circulation tumor DNA in colorectal cancer patients. Cancer Invest (2020) 38(2):85–93. 31939681 10.1080/07357907.2020.1713350

[26] Cohen JD Li L Wang Y Thoburn C Afsari B Danilova L. Detection and localization of surgically resectable cancers with a multi-analyte blood test. Science (2018) 359(6378):926–30. 10.1126/science.aar3247 PMC6080308 29348365

[27] Phallen J Sausen M Adleff V Leal A Hruban C White J. Direct detection of early-stage cancers using circulating tumor DNA. Sci Transl Med (2017) 9(403). 10.1126/scitranslmed.aan2415 PMC6714979 28814544

[28] Tzanikou E Lianidou E. The potential of ctDNA analysis in breast cancer. Crit Rev Clin Lab Sci (2020) 57(1):54–72:eaan2415. 31674269 10.1080/10408363.2019.1670615

[29] Liu L Toung JM Jassowicz AF Vijayaraghavan R Kang H Zhang R. Targeted methylation sequencing of plasma cell-free DNA for cancer detection and classification. Ann Oncol (2018) 29(6):1445–53. 10.1093/annonc/mdy119 PMC6005020 29635542

[30] Shan M Yin H Li J Li X Wang D Su Y. Detection of aberrant methylation of a six-gene panel in serum DNA for diagnosis of breast cancer. Oncotarget (2016) 7(14):18485–94. 10.18632/oncotarget.7608 PMC4951303 26918343

[31] Liu X Lang J Li S Wang Y Peng L Wang W. Fragment enrichment of circulating tumor DNA with low-frequency mutations. Front Genet (2020) 11:147. 32180799 10.3389/fgene.2020.00147 PMC7059766

[32] Jiang P Chan CW Chan KC Cheng SH Wong J Wong VW. Lengthening and shortening of plasma DNA in hepatocellular carcinoma patients. Proc Natl Acad Sci USA (2015) 112(11):E1317–25. 10.1073/pnas.1500076112 PMC4372002 25646427

[33] Cristiano S Leal A Phallen J Fiksel J Adleff V Bruhm DC. Genome-wide cell-free DNA fragmentation in patients with cancer. Nature (2019) 570(7761):385–9. 10.1038/s41586-019-1272-6 PMC6774252 31142840

[34] Snyder MW Kircher M Hill AJ Daza RM Shendure J. Cell-free DNA comprises an in vivo nucleosome footprint that informs its tissues-Of-Origin. Cell (2016) 164(1-2):57–68. 26771485 10.1016/j.cell.2015.11.050 PMC4715266

[35] Sun K Jiang P Cheng SH Cheng THT Wong J Wong VWS. Orientation-aware plasma cell-free DNA fragmentation analysis in open chromatin regions informs tissue of origin. Genome Res (2019) 29(3):418–27. 10.1101/gr.242719.118 PMC6396422 30808726

[36] Han DSC Lo YMD. The nexus of cfDNA and nuclease biology. Trends Genet (2021) 37(8):758–70. 10.1016/j.tig.2021.04.005 34006390

[37] Serpas L Chan RWY Jiang P Ni M Sun K Rashidfarrokhi A. Dnase1l3 deletion causes aberrations in length and end-motif frequencies in plasma DNA. Proc Natl Acad Sci USA (2019) 116(2):641–9. 10.1073/pnas.1815031116 PMC6329986 30593563

[38] Jiang P Sun K Peng W Cheng SH Ni M Yeung PC. Plasma DNA end-motif profiling as a fragmentomic marker in cancer, pregnancy, and transplantation. Cancer Discovery (2020) 10(5):664–73. 10.1158/2159-8290.CD-19-0622 32111602

[39] Shao X Lv N Liao J Long J Xue R Ai N. Copy number variation is highly correlated with differential gene expression: a pan-cancer study. BMC Med Genet (2019) 20(1):175. 31706287 10.1186/s12881-019-0909-5 PMC6842483

[40] Meng Z Ren Q Zhong G Li S Chen Y Wu W. Noninvasive detection of hepatocellular carcinoma with circulating tumor DNA features and α-fetoprotein. J Mol Diagn (2021) 23(9):1174–84. 10.1016/j.jmoldx.2021.06.003 34182124

[41] Nguyen HT Khoa Huynh LA Nguyen TV Tran DH Thu Tran TT Khang Le ND. Multimodal analysis of ctDNA methylation and fragmentomic profiles enhances detection of nonmetastatic colorectal cancer. Future Oncol (2022) 18(35):3895–912. 10.2217/fon-2022-1041 36524960

[42] Mouliere F Chandrananda D Piskorz AM Moore EK Morris J Ahlborn LB. Enhanced detection of circulating tumor DNA by fragment size analysis. Sci Transl Med (2018) 10(466):eaat4921. 30404863 10.1126/scitranslmed.aat4921 PMC6483061

[43] Wei Q Jin C Wang Y Guo S Guo X Liu X. A computational framework to unify orthogonal information in DNA methylation and copy number aberrations in cell-free DNA for early cancer detection. Brief Bioinform (2022) 23(4):bbac200. 35649341 10.1093/bib/bbac200

[44] Edge SB Compton CC. The American joint committee on cancer: the 7th edition of the AJCC cancer staging manual and the future of TNM. Ann Surg Oncol (2010) 17(6):1471–4. 10.1245/s10434-010-0985-4 20180029

[45] Guiu S Michiels S André F Cortes J Denkert C Di Leo A. Molecular subclasses of breast cancer: how do we define them? the IMPAKT 2012 working group statement. Ann Oncol (2012) 23(12):2997–3006. 23166150 10.1093/annonc/mds586

[46] Jiang P Sun K Lun FM Guo AM Wang H Chan KC. Methy-pipe: an integrated bioinformatics pipeline for whole genome bisulfite sequencing data analysis. PloS One (2014) 9(6):e100360. 24945300 10.1371/journal.pone.0100360 PMC4063866

[47] Scheinin I Sie D Bengtsson H van de Wiel MA Olshen AB van Thuijl HF. DNA Copy number analysis of fresh and formalin-fixed specimens by shallow whole-genome sequencing with identification and exclusion of problematic regions in the genome assembly. Genome Res (2014) 24(12):2022–32. 10.1101/gr.175141.114 PMC4248318 25236618

[48] Amemiya HM Kundaje A Boyle AP. The ENCODE blacklist: identification of problematic regions of the genome. Sci Rep (2019) 9(1):9354. 31249361 10.1038/s41598-019-45839-z PMC6597582

[49] DeLong ER DeLong DM Clarke-Pearson DL. Comparing the areas under two or more correlated receiver operating characteristic curves: a nonparametric approach. Biometrics (1988) 44(3):837–45. 3203132

[50] Underhill HR Kitzman JO Hellwig S Welker NC Daza R Baker DN. Fragment length of circulating tumor DNA. PloS Genet (2016) 12(7):e1006162. 27428049 10.1371/journal.pgen.1006162 PMC4948782

[51] Hallermayr A Wohlfrom T Steinke-Lange V Benet-Pagès A Scharf F Heitzer E. Somatic copy number alteration and fragmentation analysis in circulating tumor DNA for cancer screening and treatment monitoring in colorectal cancer patients. J Hematol Oncol (2022) 15(1):125. 36056434 10.1186/s13045-022-01342-z PMC9438339

[52] Nguyen VC Nguyen TH Phan TH Tran TT Pham TTT Ho TD. Fragment length profiles of cancer mutations enhance detection of circulating tumor DNA in patients with early-stage hepatocellular carcinoma. BMC Cancer (2023) 23(1):233. 36915069 10.1186/s12885-023-10681-0 PMC10009971

[53] Jin C Liu X Zheng W Su L Liu Y Guo X. Characterization of fragment sizes, copy number aberrations and 4-mer end motifs in cell-free DNA of hepatocellular carcinoma for enhanced liquid biopsy-based cancer detection. Mol Oncol (2021) 15(9):2377–89. 10.1002/1878-0261.13041 PMC8410516 34133846

[54] Chen L Abou-Alfa GK Zheng B Liu JF Bai J Du LT. Genome-scale profiling of circulating cell-free DNA signatures for early detection of hepatocellular carcinoma in cirrhotic patients. Cell Res (2021) 31(5):589–92. 10.1038/s41422-020-00457-7 PMC8089097 33589745

[55] Baldacchino S Grech G. Somatic copy number aberrations in metastatic patients: the promise of liquid biopsies. Semin Cancer Biol (2020) 60:302–10. 10.1016/j.semcancer.2019.12.014 31891778

[56] Knuutila S Aalto Y Autio K Björkqvist AM El-Rifai W Hemmer S. DNA Copy number losses in human neoplasms. Am J Pathol (1999) 155(3):683–94. 10.1016/S0002-9440(10)65166-8 PMC1866903 10487825

[57] Dereli-Öz A Versini G Halazonetis TD. Studies of genomic copy number changes in human cancers reveal signatures of DNA replication stress. Mol Oncol (2011) 5(4):308–14. 10.1016/j.molonc.2011.05.002 PMC5528318 21641882

[58] Cheng THT Jiang P Teoh JYC Heung MMS Tam JCW Sun X. Noninvasive detection of bladder cancer by shallow-depth genome-wide bisulfite sequencing of urinary cell-free DNA for methylation and copy number profiling. Clin Chem (2019) 65(7):927–36. 10.1373/clinchem.2018.301341 30988170

[59] Van Roy N van der Linden M Menten B Dheedene A Vandeputte C Van Dorpe J. Shallow whole genome sequencing on circulating cell-free DNA allows reliable noninvasive copy-number profiling in neuroblastoma patients. Clin Cancer Res (2017) 23(20):6305–14. 10.1158/1078-0432.CCR-17-0675 28710315

[60] Chan KC Jiang P Chan CW Sun K Wong J Hui EP. Noninvasive detection of cancer-associated genome-wide hypomethylation and copy number aberrations by plasma DNA bisulfite sequencing. Proc Natl Acad Sci USA (2013) 110(47):18761–8. 10.1073/pnas.1313995110 PMC3839703 24191000

[61] Hon GC Hawkins RD Caballero OL Lo C Lister R Pelizzola M. Global DNA hypomethylation coupled to repressive chromatin domain formation and gene silencing in breast cancer. Genome Res (2012) 22(2):246–58. 10.1101/gr.125872.111 PMC3266032 22156296

[62] Szyf M Pakneshan P Rabbani SA. DNA Methylation and breast cancer. Biochem Pharmacol (2004) 68(6):1187–97. 10.1016/j.bcp.2004.04.030 15313416

[63] Li Z Zhang X Hou C Zhou Y Chen J Cai H. Comprehensive identification and characterization of somatic copy number alterations in triple−negative breast cancer. Int J Oncol (2020) 56(2):522–30. 10.3892/ijo.2019.4950 PMC6959384 31894314

[64] Staaf J Jönsson G Ringnér M Baldetorp B Borg A. Landscape of somatic allelic imbalances and copy number alterations in HER2-amplified breast cancer. Breast Cancer Res (2011) 13(6):R129. 22169037 10.1186/bcr3075 PMC3326571

[65] Xu RH Wei W Krawczyk M Wang W Luo H Flagg K. Circulating tumour DNA methylation markers for diagnosis and prognosis of hepatocellular carcinoma. Nat Mater (2017) 16(11):1155–61. 10.1038/nmat4997 29035356

[66] Das PM Singal R. DNA Methylation and cancer. J Clin Oncol (2004) 22(22):4632–42. 10.1200/JCO.2004.07.151 15542813

[67] Ehrlich M. DNA Methylation in cancer: too much, but also too little. Oncogene (2002) 21(35):5400–13. 10.1038/sj.onc.1205651 12154403

[68] Ehrlich M. DNA Hypomethylation in cancer cells. Epigenomics (2009) 1(2):239–59. 10.2217/epi.09.33 PMC2873040 20495664

[69] Hu CY Mohtat D Yu Y Ko YA Shenoy N Bhattacharya S. Kidney cancer is characterized by aberrant methylation of tissue-specific enhancers that are prognostic for overall survival. Clin Cancer Res (2014) 20(16):4349–60. 10.1158/1078-0432.CCR-14-0494 PMC4424050 24916699

[70] Ennour-Idrissi K Dragic D Durocher F Diorio C. Epigenome-wide DNA methylation and risk of breast cancer: a systematic review. BMC Cancer (2020) 20(1):1048. 33129307 10.1186/s12885-020-07543-4 PMC7603741

[71] van Veldhoven K Polidoro S Baglietto L Severi G Sacerdote C Panico S. Epigenome-wide association study reveals decreased average methylation levels years before breast cancer diagnosis. Clin Epigenet (2015) 7(1):67. 10.1186/s13148-015-0104-2 PMC4524428 26244061

[72] Bernardino J Roux C Almeida A Vogt N Gibaud A Gerbault-Seureau M. DNA Hypomethylation in breast cancer: an independent parameter of tumor progression? Cancer Genet Cytogenet (1997) 97(2):83–9. 10.1016/s0165-4608(96)00385-8 9283586

[73] Kar S Sengupta D Deb M Shilpi A Parbin S Rath SK. Expression profiling of DNA methylation-mediated epigenetic gene-silencing factors in breast cancer. Clin Epigenet (2014) 6(1):20. 10.1186/1868-7083-6-20 PMC4255691 25478034

[74] Man X Li Q Wang B Zhang H Zhang S Li Z. DNMT3A and DNMT3B in breast tumorigenesis and potential therapy. Front Cell Dev Biol (2022) 10. 10.3389/fcell.2022.916725 PMC9127442 35620052

[75] Herman JG. Hypermethylation of tumor suppressor genes in cancer. Semin Cancer Biol (1999) 9(5):359–67. 10.1006/scbi.1999.0138 10547344

[76] Ivanov M Baranova A Butler T Spellman P Mileyko V. Non-random fragmentation patterns in circulating cell-free DNA reflect epigenetic regulation. BMC Genomics (2015) 16 Suppl 13(Suppl 13):S1. 10.1186/1471-2164-16-S13-S1 PMC4686799 26693644

