# Supplementary material for: Multimodal analysis of genome-wide methylation, copy number aberrations, and end motif signatures enhances detection of early-stage breast cancer
Source: Front Oncol. 2023 May 8;13:1127086. doi: 10.3389/fonc.2023.1127086 (PMC10200909; doi:10.3389/fonc.2023.1127086)
Supplement: Supplementary file 1 [file DataSheet_1.pdf]

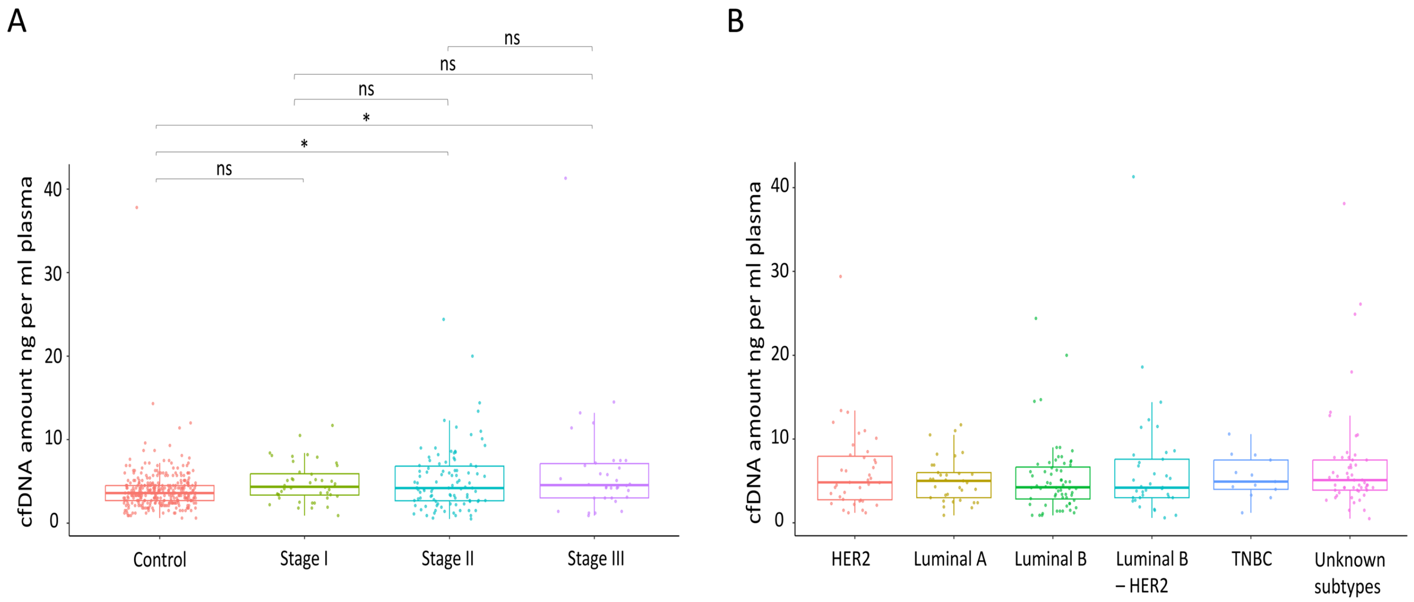

Figure S1. cfDNA concentrations of breast cancer patients and healthy subjects.

(A) Box plots showing the concentrations of cfDNA (ng/ml plasma) isolated from healthy individuals or breast cancer patients with different tumor stages. (B) Box plots showing the concentrations of cfDNA (ng/ml plasma) isolated from breast cancer patients with different subtypes. *, $p<0.05$; **, $p<0.001$; ns, not significant (Kruskal-Wallis test).

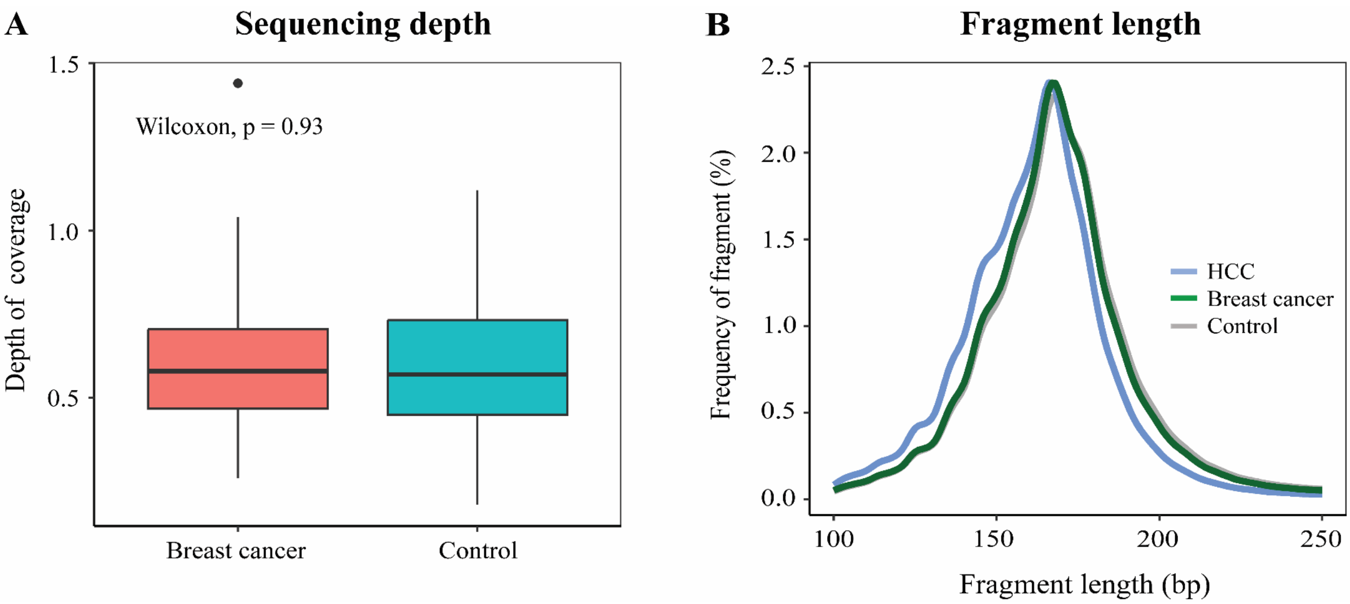

Figure S2: (A) Boxplots displayed whole-genome sequencing depth for breast cancer (red) and control (blue). (B) Density curves displayed fragment length distribution of HCC (light-blue), breast cancer (dark-green), and control (grey).

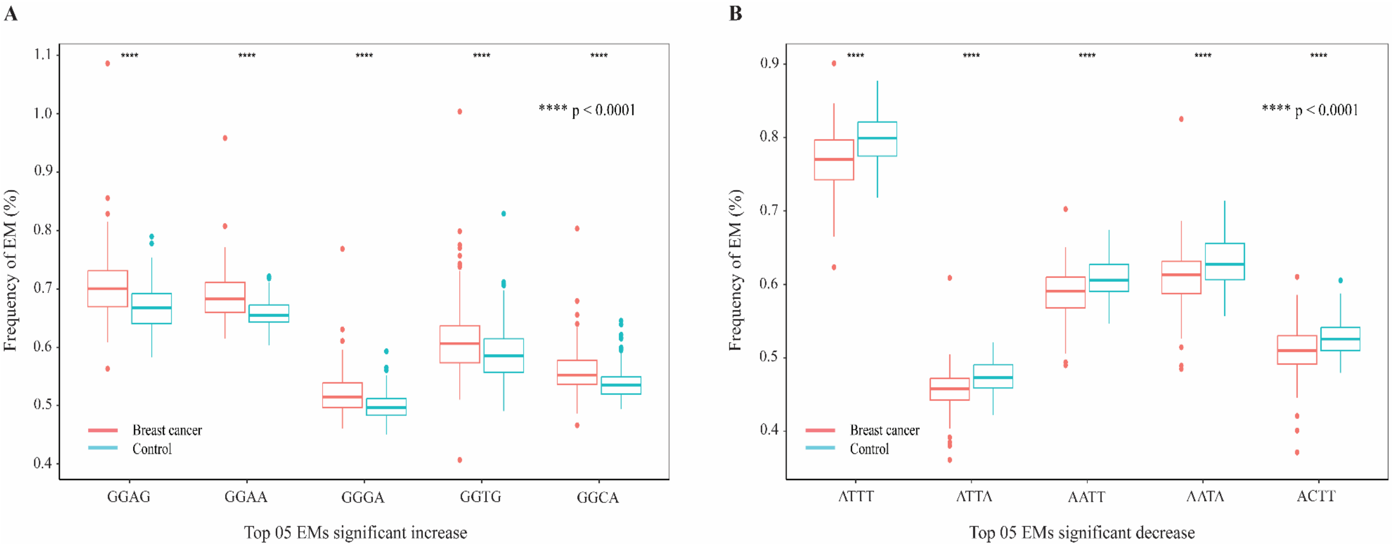

Figure S3: Boxplots displayed frequencies of top five EMs with significant frequency increase (A) and significant frequency decrease (B) in breast cancer compared to control.

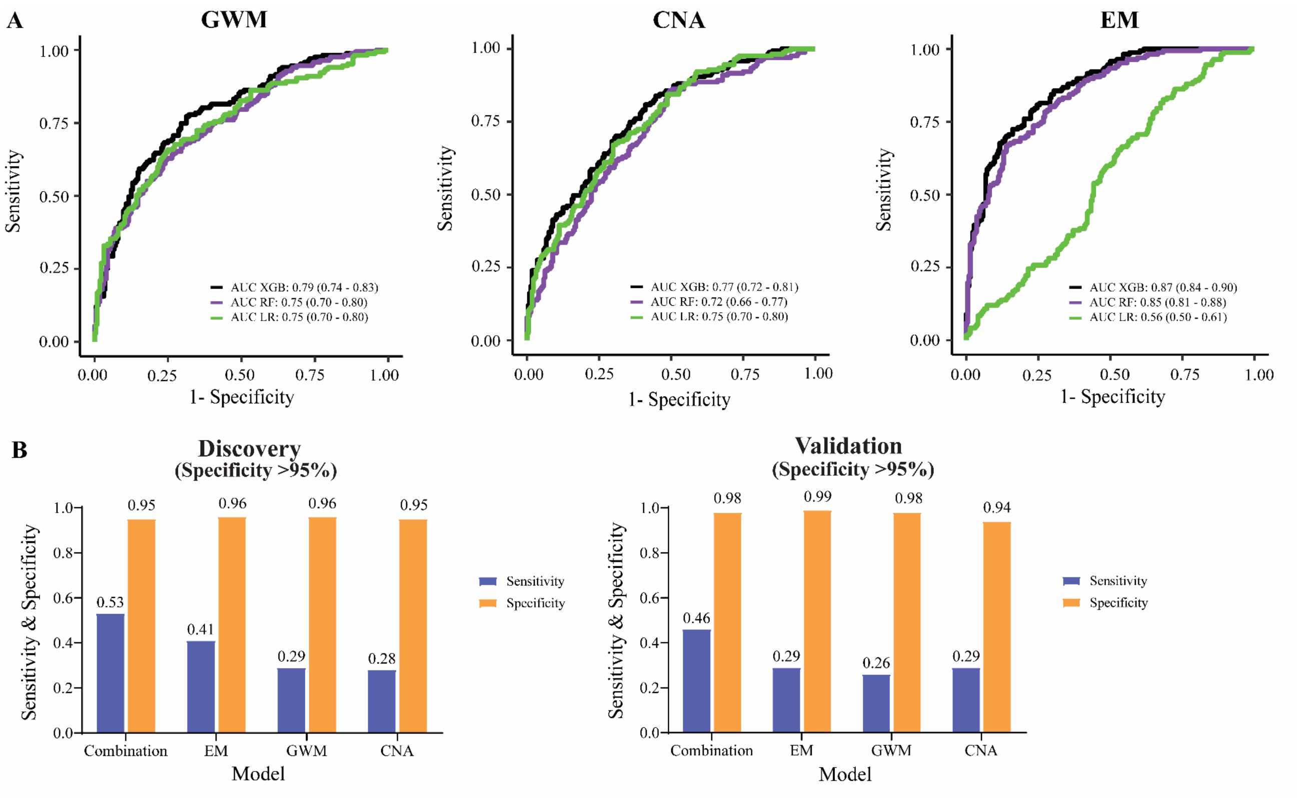

Figure S4: (A) ROC curves showed performance of XGB (black), RF (purple) and LR (green) algorithm with GWM, CNA and EM feature each as input. (B) Bar graphs displayed sensitivity and specificity, when specificity was set as at least 95%, for the combination and individual-feature models in the discovery and validation cohort.

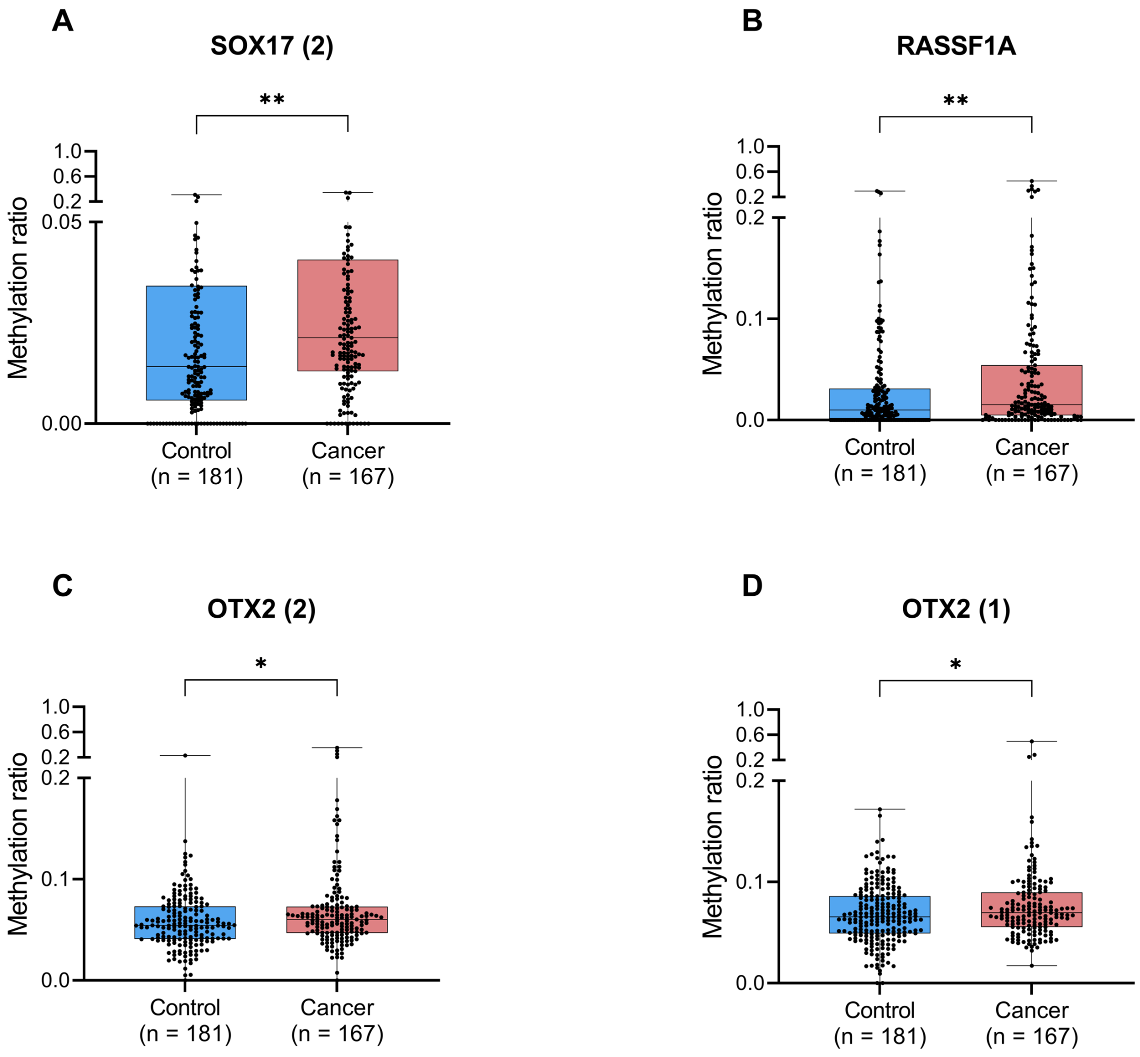

Figure S5. Methylation levels of significant DMRs between breast cancer and healthy subjects. (A)- (D) Box plot showing methylation levels of the 4 DMRs with p-value <0.05 , differentiating breast cancer patients (n=181) from healthy individuals (n=167) in the discovery cohort.*, p<0.05; **, p<0.001 (Wilcoxon Rank Sum test).
