# Supplementary material for: Multimodal analysis of genome-wide methylation, copy number aberrations, and end motif signatures enhances detection of early-stage breast cancer
Source: Front Oncol. 2023 May 8;13:1127086. doi: 10.3389/fonc.2023.1127086 (PMC10200909; doi:10.3389/fonc.2023.1127086)
Supplement: Supplementary file 2 [file Table_1.docx]

| **Table S1.** Clinical characteristics of breast cancer patients (n= 239) and healthy control (n=278) | | | | | | | | | | | |
| --- | --- | --- | --- | --- | --- | --- | --- | --- | --- | --- | --- |
| **No** | **Group** | **Label** | **SampleID** | **Age** | **Gender** | **Tumor Stage** | **Tumor type** | **Background lesions** | | | **Subtype** |
|  |  |  |  |  |  |  |  | **Metastasis** | **Number of tumors** | **Tumor diameter** |  |
| 1 | Discovery | Breast cancer | LBMB011 | 62 | F | II | Carcinoma | Yes | 1 | 9x13mm | Luminal B - HER2 |
| 2 | Discovery | Breast cancer | LBMB043 | 56 | F | IB | Carcinoma | No | 1 | 4x6mm | Luminal A |
| 3 | Discovery | Breast cancer | LBMB200 | 61 | F | IIA | Carcinoma | No | 2 | 14x20mm, 12x24mm | HER2 |
| 4 | Discovery | Breast cancer | LBMB203 | 47 | F | NA | Carcinoma | NA | 1 | 44x22mm | NA |
| 5 | Discovery | Breast cancer | LBMB204 | 39 | F | IIA | Carcinoma | No | NA | NA | Luminal A |
| 6 | Discovery | Breast cancer | LBMB206 | 72 | F | NA | Carcinoma | NA | NA | NA | NA |
| 7 | Discovery | Breast cancer | LBMB207 | 67 | F | II | Carcinoma | No | NA | NA | Luminal B - HER2 |
| 8 | Discovery | Breast cancer | LBMB208 | 65 | F | NA | Carcinoma | NA | NA | NA | NA |
| 9 | Discovery | Breast cancer | LBMB210 | 48 | F | NA | Carcinoma | NA | NA | NA | Luminal B - HER2 |
| 10 | Discovery | Breast cancer | LBMB213 | 57 | F | NA | Carcinoma | NA | NA | NA | NA |
| 11 | Discovery | Breast cancer | LBMB215 | 53 | F | IIA | Carcinoma | No | NA | NA | HER2 |
| 12 | Discovery | Breast cancer | LBMB216 | 37 | F | NA | Carcinoma | NA | NA | NA | NA |
| 13 | Discovery | Breast cancer | LBMB217 | 51 | F | II | Carcinoma | No | 1 | 12x11mm | Luminal B |
| 14 | Discovery | Breast cancer | LBMB218 | 59 | F | NA | Carcinoma | NA | NA | NA | NA |
| 15 | Discovery | Breast cancer | LBMB220 | 39 | F | NA | Carcinoma | NA | NA | NA | NA |
| 16 | Discovery | Breast cancer | LBMB221 | 65 | F | NA | Carcinoma | NA | NA | NA | NA |
| 17 | Discovery | Breast cancer | LBMB222 | 44 | F | II | Carcinoma | NA | NA | NA | TNBC |
| 18 | Discovery | Breast cancer | LBMB223 | 47 | F | NA | Carcinoma | NA | NA | NA | Luminal B |
| 19 | Discovery | Breast cancer | LBMB225 | 58 | F | IIA | Carcinoma | NA | NA | NA | Luminal A |
| 20 | Discovery | Breast cancer | LBMB226 | 68 | F | NA | Carcinoma | NA | NA | NA | HER2 |
| 21 | Discovery | Breast cancer | LBMB230 | 57 | F | NA | Carcinoma | NA | NA | NA | NA |
| 22 | Discovery | Breast cancer | LBMB231 | 47 | F | NA | Carcinoma | NA | NA | NA | NA |
| 23 | Discovery | Breast cancer | LBMB232 | 66 | F | NA | Carcinoma | NA | NA | NA | NA |
| 24 | Discovery | Breast cancer | LBMB233 | 55 | F | NA | Carcinoma | NA | NA | NA | NA |
| 25 | Discovery | Breast cancer | LBMB235 | 47 | F | IIA | Carcinoma | NA | 1 | 22x15mm | Luminal A |
| 26 | Discovery | Breast cancer | LBMB239 | 58 | F | II | Carcinoma | No | 1 | 17x14mm | Luminal B |
| 27 | Discovery | Breast cancer | LBMB240 | 51 | F | II | Carcinoma | No | 1 | 15x22mm | Luminal B |
| 28 | Discovery | Breast cancer | LBMB241 | 67 | F | NA | Carcinoma | NA | NA | NA | NA |
| 29 | Discovery | Breast cancer | LBMB242 | 60 | F | NA | Carcinoma | NA | NA | NA | NA |
| 30 | Discovery | Breast cancer | LBMB243 | 61 | F | NA | Carcinoma | NA | NA | NA | Luminal A |
| 31 | Discovery | Breast cancer | LBMB244 | 67 | F | NA | Carcinoma | NA | NA | NA | Luminal A |
| 32 | Discovery | Breast cancer | LBMB245 | 44 | F | NA | Carcinoma | NA | NA | NA | NA |
| 33 | Discovery | Breast cancer | LBMB246 | 48 | F | II | Carcinoma | No | 1 | 2,6x4mm | HER2 |
| 34 | Discovery | Breast cancer | LBMB248 | 66 | F | NA | Carcinoma | NA | NA | NA | NA |
| 35 | Discovery | Breast cancer | LBMB249 | 48 | F | NA | Carcinoma | NA | NA | NA | NA |
| 36 | Discovery | Breast cancer | LBMB251 | 52 | F | NA | Carcinoma | NA | NA | NA | Luminal B - HER2 |
| 37 | Discovery | Breast cancer | LBMB253 | 59 | F | IIA | Carcinoma | No | 1 | 18x14mm | Luminal A |
| 38 | Discovery | Breast cancer | LBMB256 | 52 | F | NA | Carcinoma | NA | NA | NA | NA |
| 39 | Discovery | Breast cancer | LBMB260 | 41 | F | NA | Carcinoma | NA | NA | NA | NA |
| 40 | Discovery | Breast cancer | LBMB262 | 41 | F | I | Carcinoma | No | 1 | 15x7mm | Luminal A |
| 41 | Discovery | Breast cancer | LBMB263 | 36 | F | NA | Carcinoma | NA | NA | NA | NA |
| 42 | Discovery | Breast cancer | LBMB264 | 45 | F | NA | Carcinoma | NA | NA | NA | HER2 |
| 43 | Discovery | Breast cancer | LBMB265 | 49 | F | NA | Carcinoma | NA | NA | NA | NA |
| 44 | Discovery | Breast cancer | LBMB268 | 45 | F | II | Carcinoma | No | 1 | 12x17mm | Luminal B |
| 45 | Discovery | Breast cancer | LBMB269 | 50 | F | I | Carcinoma | No | 1 | 8x11mm | Luminal B - HER2 |
| 46 | Discovery | Breast cancer | LBMB270 | 53 | F | NA | Carcinoma | NA | NA | NA | NA |
| 47 | Discovery | Breast cancer | LBMB271 | 48 | F | NA | Carcinoma | NA | NA | NA | NA |
| 48 | Discovery | Breast cancer | LBMB273R2 | 51 | F | NA | Carcinoma | NA | NA | NA | HER2 |
| 49 | Discovery | Breast cancer | LBMB274 | 50 | F | I | Carcinoma | No | NA | NA | Luminal A |
| 50 | Discovery | Breast cancer | LBMB275R2 | 34 | F | NA | Carcinoma | NA | NA | NA | Luminal B |
| 51 | Discovery | Breast cancer | LBMB276R2 | 47 | F | NA | Carcinoma | NA | NA | NA | NA |
| 52 | Discovery | Breast cancer | LBMB280R2 | 62 | F | NA | Carcinoma | NA | NA | NA | NA |
| 53 | Discovery | Breast cancer | LBMB282 | 29 | F | NA | Carcinoma | NA | NA | NA | TNBC |
| 54 | Discovery | Breast cancer | LBMB285 | 62 | F | NA | Carcinoma | NA | NA | NA | NA |
| 55 | Discovery | Breast cancer | LBMB286 | 66 | F | NA | Carcinoma | NA | NA | NA | NA |
| 56 | Discovery | Breast cancer | LBMB288 | 56 | F | NA | Carcinoma | NA | NA | NA | NA |
| 57 | Discovery | Breast cancer | LBMB289 | 42 | F | NA | Carcinoma | NA | NA | NA | NA |
| 58 | Discovery | Breast cancer | LBMB290 | 54 | F | NA | Carcinoma | NA | NA | NA | NA |
| 59 | Discovery | Breast cancer | LBMB291 | 61 | F | NA | Carcinoma | NA | NA | NA | NA |
| 60 | Discovery | Breast cancer | LBMB292 | 60 | F | NA | Carcinoma | NA | NA | NA | NA |
| 61 | Discovery | Breast cancer | LBMB293 | 49 | F | NA | Carcinoma | NA | NA | NA | NA |
| 62 | Discovery | Breast cancer | LBMB294 | 72 | F | NA | Carcinoma | NA | NA | NA | NA |
| 63 | Discovery | Breast cancer | MB008 | 62 | F | I | Carcinoma | No | 1 | 7,5mm | Luminal A |
| 64 | Discovery | Breast cancer | MB009 | 63 | F | I | Carcinoma | Yes | 1 | 20x26mm | Luminal B |
| 65 | Discovery | Breast cancer | MB010AR | 56 | F | III | Carcinoma | Yes | 1 | 300mm | Luminal B |
| 66 | Discovery | Breast cancer | MB016A | 41 | F | III | Carcinoma | Yes | 1 | 21x27mm | Luminal B |
| 67 | Discovery | Breast cancer | MB017 | 55 | F | II | Carcinoma | No | 2 | 9mm, 14x21mm | Luminal B - HER2 |
| 68 | Discovery | Breast cancer | MB018R1 | 57 | F | II | Carcinoma | No | 1 | 24x14mm | Luminal B |
| 69 | Discovery | Breast cancer | MB021 | 47 | F | II | Carcinoma | No | 1 | 36x17mm | Luminal B - HER2 |
| 70 | Discovery | Breast cancer | MB023A | 40 | F | II | Carcinoma | No | 1 | 40x23mm | Luminal A |
| 71 | Discovery | Breast cancer | MB026A | 60 | F | III | Carcinoma | No | 1 | 12x28mm | HER2 |
| 72 | Discovery | Breast cancer | MB028 | 64 | F | II | Carcinoma | No | 1 | 11x23mm | Luminal B |
| 73 | Discovery | Breast cancer | MB031A | 36 | F | I | Carcinoma | No | 1 | 8x9mm | Luminal B - HER2 |
| 74 | Discovery | Breast cancer | MB032A | 39 | F | II | Carcinoma | No | 1 | 28mm | Luminal B |
| 75 | Discovery | Breast cancer | MB037A | 54 | F | II | Carcinoma | No | 1 | 26x13mm | Luminal B - HER2 |
| 76 | Discovery | Breast cancer | MB038A | 48 | F | II | Carcinoma | No | 1 | 2,6x4mm | HER2 |
| 77 | Discovery | Breast cancer | MB040 | 50 | F | II | Carcinoma | No | 1 | 22x17mm | TNBC |
| 78 | Discovery | Breast cancer | MB043 | 55 | F | II | Carcinoma | No | 1 | 26x17mm | HER2 |
| 79 | Discovery | Breast cancer | MB046A | 55 | F | I | Carcinoma | No | 1 | 19x13,2mm | Luminal A |
| 80 | Discovery | Breast cancer | MB047A | 77 | F | II | Carcinoma | No | 1 | 35x23mm | Luminal B |
| 81 | Discovery | Breast cancer | MB048A | 47 | F | II | Carcinoma | No | 1 | 35x15mm | HER2 |
| 82 | Discovery | Breast cancer | MB049A | 44 | F | II | Carcinoma | No | 1 | 26x18x42mm | HER2 |
| 83 | Discovery | Breast cancer | MB050A | 48 | F | II | Carcinoma | No | 1 | 24x18mm | Luminal B |
| 84 | Discovery | Breast cancer | MB054A | 41 | F | II | Carcinoma | Yes | 1 | 32x19mm | HER2 |
| 85 | Discovery | Breast cancer | MB055A | 56 | F | I | Carcinoma | No | 1 | 15x13mm | TNBC |
| 86 | Discovery | Breast cancer | MB056AR | 37 | F | II | Carcinoma | No | 1 | 24x34mm | TNBC |
| 87 | Discovery | Breast cancer | MB058A | 66 | F | I | Carcinoma | No | 1 | 5x9mm | HER2 |
| 88 | Discovery | Breast cancer | MB059AR | 64 | F | III | Carcinoma | Yes | 2 | 30x20mm, 13mm | HER2 |
| 89 | Discovery | Breast cancer | MB063A | 60 | F | III | Carcinoma | Yes | 1 | 25mm | Luminal B - HER2 |
| 90 | Discovery | Breast cancer | MB064A | 59 | F | I | Carcinoma | Yes | 1 | 12x14mm | Luminal A |
| 91 | Discovery | Breast cancer | MB065 | 61 | F | II | Carcinoma | No | 1 | 30x20mm | HER2 |
| 92 | Discovery | Breast cancer | MB066A | 52 | F | I | Carcinoma | No | 1 | 14x8mm | Luminal B |
| 93 | Discovery | Breast cancer | MB067A | 59 | F | II | Carcinoma | No | 1 | 24x18mm | Luminal A |
| 94 | Discovery | Breast cancer | MB068 | 38 | F | II | Carcinoma | Yes | 1 | 35x22mm | Luminal B - HER2 |
| 95 | Discovery | Breast cancer | MB070 | 68 | F | II | Carcinoma | No | 1 | 24x14mm | Luminal A |
| 96 | Discovery | Breast cancer | MB072A | 68 | F | II | Carcinoma | No | 1 | 24x18mm | TNBC |
| 97 | Discovery | Breast cancer | MB075A | 38 | F | III | Carcinoma | Yes | 1 | 13x16mm | Luminal B |
| 98 | Discovery | Breast cancer | MB078A | 66 | F | II | Carcinoma | No | 1 | 35mm | Luminal B |
| 99 | Discovery | Breast cancer | MB079AR | 44 | F | II | Carcinoma | No | 1 | 23x19x22mm | Luminal B |
| 100 | Discovery | Breast cancer | MB081AR | 45 | F | I | Carcinoma | Yes | 1 | 12x5mm | Luminal B |
| 101 | Discovery | Breast cancer | MB084AR | 52 | F | II | Carcinoma | Yes | 1 | 24x19mm | HER2 |
| 102 | Discovery | Breast cancer | MB085A | 44 | F | I | Carcinoma | No | 1 | 14x16mm | Luminal B |
| 103 | Discovery | Breast cancer | MB086AR | 61 | F | III | Carcinoma | Yes | 1 | 15x18mm | Luminal B - HER2 |
| 104 | Discovery | Breast cancer | MB087 | 63 | F | II | Carcinoma | No | 1 | 28x18mm | Luminal B |
| 105 | Discovery | Breast cancer | MB088AR | 57 | F | III | Carcinoma | Yes | 1 | 25x15mm | Luminal B - HER2 |
| 106 | Discovery | Breast cancer | MB089A | 63 | F | II | Carcinoma | No | 1 | 20mm | Luminal B |
| 107 | Discovery | Breast cancer | MB091 | 44 | F | II | Carcinoma | No | 1 | 10x23mm | Luminal A |
| 108 | Discovery | Breast cancer | MB093 | 41 | F | I | Carcinoma | Yes | 1 | 15x15mm | Luminal A |
| 109 | Discovery | Breast cancer | MB094 | 51 | F | I | Carcinoma | No | 1 | 28x12mm | Luminal B |
| 110 | Discovery | Breast cancer | MB095A | 43 | F | III | Carcinoma | No | 2 | 40x36mm, 20x8mm | TNBC |
| 111 | Discovery | Breast cancer | MB096 | 80 | F | I | Carcinoma | No | 2 | 7x10mm, 19x16mm | Luminal A |
| 112 | Discovery | Breast cancer | MB100 | 43 | F | III | Carcinoma | Yes | 1 | 5cm | Luminal B |
| 113 | Discovery | Breast cancer | MB102 | 62 | F | II | Carcinoma | Yes | 1 | 22x21x20mm | Luminal B - HER2 |
| 114 | Discovery | Breast cancer | MB108A | 61 | F | NA | Carcinoma | Yes | 1 | 8cm | HER2 |
| 115 | Discovery | Breast cancer | MB110 | 47 | F | IIIA | Carcinoma | Yes | 1 | 13x29mm | TNBC |
| 116 | Discovery | Breast cancer | MB114A | 48 | F | I | Carcinoma | NA | 1 | 15x18mm | Luminal B - HER2 |
| 117 | Discovery | Breast cancer | MB115 | 43 | F | IIIA | Carcinoma | Yes | 1 | 20x21mm | Luminal B - HER2 |
| 118 | Discovery | Breast cancer | MB116 | 25 | F | IIA | Carcinoma | No | 1 | 2cm | Luminal B - HER2 |
| 119 | Discovery | Breast cancer | MB117 | 34 | F | IIA | Carcinoma | Yes | 1 | 3cm | HER2 |
| 120 | Discovery | Breast cancer | MB118 | 56 | F | II | Carcinoma | Yes | 1 | 2cm | Luminal B - HER2 |
| 121 | Discovery | Breast cancer | MB119 | 44 | F | II | Carcinoma | No | 1 | 5x4cm | Luminal B |
| 122 | Discovery | Breast cancer | MB122 | 65 | F | IIA | Carcinoma | No | 1 | 16x24mm | Luminal B |
| 123 | Discovery | Breast cancer | MB123 | 77 | F | IIB | Carcinoma | Yes | 1 | 22x27x26mm | Luminal B |
| 124 | Discovery | Breast cancer | MB124 | 37 | F | IIA | Carcinoma | Yes | 1 | 18x20x23mm | Luminal B - HER2 |
| 125 | Discovery | Breast cancer | MB129 | 32 | F | IIA | Carcinoma | No | 2 | 28x18mm, 8x6mm | Luminal B |
| 126 | Discovery | Breast cancer | MB131 | 38 | F | IIA | Carcinoma | NA | 1 | 28x19x28mm | Luminal A |
| 127 | Discovery | Breast cancer | MB132 | 59 | F | IIA | Carcinoma | Yes | 1 | 17x21x22mm | Luminal B |
| 128 | Discovery | Breast cancer | MB135 | 28 | F | IIA | Carcinoma | No | 1 | 19x33x36mm | Luminal B |
| 129 | Discovery | Breast cancer | MB138 | 52 | F | IIB | Carcinoma | NA | 1 | 2x3cm | Luminal B - HER2 |
| 130 | Discovery | Breast cancer | MB139 | 43 | F | IIA | Carcinoma | NA | 1 | 3cm | Luminal B - HER2 |
| 131 | Discovery | Breast cancer | MB144 | 42 | F | IIB | Carcinoma | NA | 1 | 36x24x51mm | Luminal B |
| 132 | Discovery | Breast cancer | MB145 | 40 | F | NA | Carcinoma | Yes | 1 | 26x26x27mm | NA |
| 133 | Discovery | Breast cancer | MB146 | 37 | F | IIIA | Carcinoma | Yes | 1 | 47x35x35mm | Luminal A |
| 134 | Discovery | Breast cancer | MB148 | 67 | F | IA | Carcinoma | NA | 1 | 1,5cm | Luminal B |
| 135 | Discovery | Breast cancer | MB152 | 41 | F | IIA | Carcinoma | NA | 2 | 3cm | Luminal A |
| 136 | Discovery | Breast cancer | MB153 | 70 | F | NA | Carcinoma | Yes | 1 | 3x3cm | HER2 |
| 137 | Discovery | Breast cancer | MB154 | 61 | F | IIA | Carcinoma | Yes | 1 | 22x11mm | Luminal B - HER2 |
| 138 | Discovery | Breast cancer | MB155 | 58 | F | II | Carcinoma | NA | 1 | 23x24x27mm | NA |
| 139 | Discovery | Breast cancer | MB161 | 37 | F | I | Carcinoma | NA | 1 | 9x11mm | NA |
| 140 | Discovery | Breast cancer | MB170 | 42 | F | II | Carcinoma | NA | 1 | 30x30x20mm | NA |
| 141 | Discovery | Breast cancer | MB171 | 48 | F | I | Carcinoma | NA | 1 | 12x6mm | NA |
| 142 | Discovery | Breast cancer | MB203 | 57 | F | II | Carcinoma | Yes | 1 | 22x13 mm | Luminal B |
| 143 | Discovery | Breast cancer | MB204AR | 60 | F | I | Carcinoma | No | 1 | 10x14mm | TNBC |
| 144 | Discovery | Breast cancer | MB206 | 33 | F | I | Carcinoma | No | 2 | 15x8 mm, 8x6 mm | Luminal B |
| 145 | Discovery | Breast cancer | MB208AR | 71 | F | I | Carcinoma | No | 1 | 10,5x10mm | Luminal A |
| 146 | Discovery | Breast cancer | MB209 | 42 | F | I | Carcinoma | No | 1 | 15x11mm | Luminal B - HER2 |
| 147 | Discovery | Breast cancer | MB210 | 38 | F | III | Carcinoma | No | 1 | 50x30mm | Luminal A |
| 148 | Discovery | Breast cancer | MB212AR | 67 | F | II | Carcinoma | Yes | 1 | 19x27mm | Luminal B - HER2 |
| 149 | Discovery | Breast cancer | MB215 | 39 | F | I | Carcinoma | No | 1 | 19x18mm | TNBC |
| 150 | Discovery | Breast cancer | NB023 | 37 | F | I | Carcinoma | No | 1 | 16mm | Luminal B |
| 151 | Discovery | Breast cancer | NB027 | 50 | F | II | Carcinoma | No | 1 | 23x21mm | HER2 |
| 152 | Discovery | Breast cancer | NB028 | 41 | F | II | Carcinoma | No | 1 | 13x22mm | HER2 |
| 153 | Discovery | Breast cancer | NB030 | 37 | F | III | Carcinoma | Yes | 1 | 20x30mm | Luminal B - HER2 |
| 154 | Discovery | Breast cancer | NB031 | 43 | F | I | Carcinoma | No | 1 | 13x9mm | Luminal A |
| 155 | Discovery | Breast cancer | NB037 | 59 | F | III | Carcinoma | Yes | 1 | 22x25mm | Luminal B - HER2 |
| 156 | Discovery | Breast cancer | NB04 | 41 | F | II | Carcinoma | No | 1 | 26mm | Luminal B - HER2 |
| 157 | Discovery | Breast cancer | NB041 | 41 | F | III | Carcinoma | Yes | 1 | 24x10x13mm | Luminal B - HER2 |
| 158 | Discovery | Breast cancer | NB045 | 64 | F | I | Carcinoma | No | 1 | 5mm | Luminal A |
| 159 | Discovery | Breast cancer | NB05 | 71 | F | I | Carcinoma | No | 1 | 1cm | HER2 |
| 160 | Discovery | Breast cancer | NB11 | 52 | F | III | Carcinoma | Yes | 1 | 25x18mm | Luminal B - HER2 |
| 161 | Discovery | Breast cancer | NB12R2 | 45 | F | II | Carcinoma | No | 1 | 22x17mm | TNBC |
| 162 | Discovery | Breast cancer | NB13 | 52 | F | II | Carcinoma | No | 1 | 25mm | Luminal B |
| 163 | Discovery | Breast cancer | NB14 | 48 | F | I | Carcinoma | No | 1 | 27mm | Luminal B |
| 164 | Discovery | Breast cancer | NB15 | 50 | F | III | Carcinoma | Yes | 1 | 10x6mm | HER2 |
| 165 | Discovery | Breast cancer | NB16 | 32 | F | III | Carcinoma | Yes | 1 | 14x7mm | Luminal B |
| 166 | Discovery | Breast cancer | NB17 | 40 | F | II | Carcinoma | No | 1 | 21x14mm | Luminal B |
| 167 | Discovery | Breast cancer | NB46 | 62 | F | I | Carcinoma | No | 1 | 20x15mm | Luminal B - HER2 |
| 168 | Discovery | Healthy control | K0001 | 54 | F | NA | NA | NA | NA | NA | NA |
| 169 | Discovery | Healthy control | K0011 | 42 | F | NA | NA | NA | NA | NA | NA |
| 170 | Discovery | Healthy control | K0013 | 56 | F | NA | NA | NA | NA | NA | NA |
| 171 | Discovery | Healthy control | K0038 | 56 | F | NA | NA | NA | NA | NA | NA |
| 172 | Discovery | Healthy control | K0040 | 40 | F | NA | NA | NA | NA | NA | NA |
| 173 | Discovery | Healthy control | K0054 | 52 | F | NA | NA | NA | NA | NA | NA |
| 174 | Discovery | Healthy control | K0058 | 44 | F | NA | NA | NA | NA | NA | NA |
| 175 | Discovery | Healthy control | K0065 | 42 | F | NA | NA | NA | NA | NA | NA |
| 176 | Discovery | Healthy control | K0087 | 40 | F | NA | NA | NA | NA | NA | NA |
| 177 | Discovery | Healthy control | K0094 | 47 | F | NA | NA | NA | NA | NA | NA |
| 178 | Discovery | Healthy control | K0099 | 41 | F | NA | NA | NA | NA | NA | NA |
| 179 | Discovery | Healthy control | K0103 | 67 | F | NA | NA | NA | NA | NA | NA |
| 180 | Discovery | Healthy control | K0109 | 53 | F | NA | NA | NA | NA | NA | NA |
| 181 | Discovery | Healthy control | K0115 | 62 | F | NA | NA | NA | NA | NA | NA |
| 182 | Discovery | Healthy control | K0122 | 64 | F | NA | NA | NA | NA | NA | NA |
| 183 | Discovery | Healthy control | K0126 | 41 | F | NA | NA | NA | NA | NA | NA |
| 184 | Discovery | Healthy control | K0131 | 45 | F | NA | NA | NA | NA | NA | NA |
| 185 | Discovery | Healthy control | K0136 | 45 | F | NA | NA | NA | NA | NA | NA |
| 186 | Discovery | Healthy control | K0143 | 52 | F | NA | NA | NA | NA | NA | NA |
| 187 | Discovery | Healthy control | K0146 | 60 | F | NA | NA | NA | NA | NA | NA |
| 188 | Discovery | Healthy control | K0148 | 48 | F | NA | NA | NA | NA | NA | NA |
| 189 | Discovery | Healthy control | K0151 | 54 | F | NA | NA | NA | NA | NA | NA |
| 190 | Discovery | Healthy control | K0164 | 56 | F | NA | NA | NA | NA | NA | NA |
| 191 | Discovery | Healthy control | K0165 | 58 | F | NA | NA | NA | NA | NA | NA |
| 192 | Discovery | Healthy control | K0167 | 75 | F | NA | NA | NA | NA | NA | NA |
| 193 | Discovery | Healthy control | K0169 | 50 | F | NA | NA | NA | NA | NA | NA |
| 194 | Discovery | Healthy control | K0172 | 41 | F | NA | NA | NA | NA | NA | NA |
| 195 | Discovery | Healthy control | K0176 | 44 | F | NA | NA | NA | NA | NA | NA |
| 196 | Discovery | Healthy control | K0178 | 45 | F | NA | NA | NA | NA | NA | NA |
| 197 | Discovery | Healthy control | K0184 | 42 | F | NA | NA | NA | NA | NA | NA |
| 198 | Discovery | Healthy control | K0185 | 50 | F | NA | NA | NA | NA | NA | NA |
| 199 | Discovery | Healthy control | K0198 | 54 | F | NA | NA | NA | NA | NA | NA |
| 200 | Discovery | Healthy control | K0201 | 49 | F | NA | NA | NA | NA | NA | NA |
| 201 | Discovery | Healthy control | K0206 | 53 | F | NA | NA | NA | NA | NA | NA |
| 202 | Discovery | Healthy control | K0207 | 54 | F | NA | NA | NA | NA | NA | NA |
| 203 | Discovery | Healthy control | K0215 | 40 | F | NA | NA | NA | NA | NA | NA |
| 204 | Discovery | Healthy control | K0229 | 52 | F | NA | NA | NA | NA | NA | NA |
| 205 | Discovery | Healthy control | K0233 | 43 | F | NA | NA | NA | NA | NA | NA |
| 206 | Discovery | Healthy control | K0244 | 41 | F | NA | NA | NA | NA | NA | NA |
| 207 | Discovery | Healthy control | K0257 | 47 | F | NA | NA | NA | NA | NA | NA |
| 208 | Discovery | Healthy control | K0258 | 64 | F | NA | NA | NA | NA | NA | NA |
| 209 | Discovery | Healthy control | K0259 | 58 | F | NA | NA | NA | NA | NA | NA |
| 210 | Discovery | Healthy control | K0271 | 54 | F | NA | NA | NA | NA | NA | NA |
| 211 | Discovery | Healthy control | K0273 | 52 | F | NA | NA | NA | NA | NA | NA |
| 212 | Discovery | Healthy control | K0278 | 42 | F | NA | NA | NA | NA | NA | NA |
| 213 | Discovery | Healthy control | K0285 | 71 | F | NA | NA | NA | NA | NA | NA |
| 214 | Discovery | Healthy control | K0286 | 45 | F | NA | NA | NA | NA | NA | NA |
| 215 | Discovery | Healthy control | K0294 | 42 | F | NA | NA | NA | NA | NA | NA |
| 216 | Discovery | Healthy control | K0405 | 43 | F | NA | NA | NA | NA | NA | NA |
| 217 | Discovery | Healthy control | K0410 | 52 | F | NA | NA | NA | NA | NA | NA |
| 218 | Discovery | Healthy control | K0412 | 45 | F | NA | NA | NA | NA | NA | NA |
| 219 | Discovery | Healthy control | K0426 | 46 | F | NA | NA | NA | NA | NA | NA |
| 220 | Discovery | Healthy control | K0431 | 47 | F | NA | NA | NA | NA | NA | NA |
| 221 | Discovery | Healthy control | K0432 | 63 | F | NA | NA | NA | NA | NA | NA |
| 222 | Discovery | Healthy control | K0444 | 42 | F | NA | NA | NA | NA | NA | NA |
| 223 | Discovery | Healthy control | K0445 | 43 | F | NA | NA | NA | NA | NA | NA |
| 224 | Discovery | Healthy control | K0447 | 43 | F | NA | NA | NA | NA | NA | NA |
| 225 | Discovery | Healthy control | K0449 | 43 | F | NA | NA | NA | NA | NA | NA |
| 226 | Discovery | Healthy control | K0453 | 56 | F | NA | NA | NA | NA | NA | NA |
| 227 | Discovery | Healthy control | K0457 | 47 | F | NA | NA | NA | NA | NA | NA |
| 228 | Discovery | Healthy control | K0460 | 54 | F | NA | NA | NA | NA | NA | NA |
| 229 | Discovery | Healthy control | K0465 | 60 | F | NA | NA | NA | NA | NA | NA |
| 230 | Discovery | Healthy control | K0467 | 44 | F | NA | NA | NA | NA | NA | NA |
| 231 | Discovery | Healthy control | K0478 | 50 | F | NA | NA | NA | NA | NA | NA |
| 232 | Discovery | Healthy control | K0483 | 65 | F | NA | NA | NA | NA | NA | NA |
| 233 | Discovery | Healthy control | K0534 | 46 | F | NA | NA | NA | NA | NA | NA |
| 234 | Discovery | Healthy control | K0568 | 60 | F | NA | NA | NA | NA | NA | NA |
| 235 | Discovery | Healthy control | K0578 | 51 | F | NA | NA | NA | NA | NA | NA |
| 236 | Discovery | Healthy control | K0590 | 67 | F | NA | NA | NA | NA | NA | NA |
| 237 | Discovery | Healthy control | K0591 | 52 | F | NA | NA | NA | NA | NA | NA |
| 238 | Discovery | Healthy control | K0597 | 46 | F | NA | NA | NA | NA | NA | NA |
| 239 | Discovery | Healthy control | K0601 | 46 | F | NA | NA | NA | NA | NA | NA |
| 240 | Discovery | Healthy control | K0610 | 55 | F | NA | NA | NA | NA | NA | NA |
| 241 | Discovery | Healthy control | K0611 | 52 | F | NA | NA | NA | NA | NA | NA |
| 242 | Discovery | Healthy control | K0616 | 47 | F | NA | NA | NA | NA | NA | NA |
| 243 | Discovery | Healthy control | K0618 | 44 | F | NA | NA | NA | NA | NA | NA |
| 244 | Discovery | Healthy control | K0619 | 43 | F | NA | NA | NA | NA | NA | NA |
| 245 | Discovery | Healthy control | K0638 | 43 | F | NA | NA | NA | NA | NA | NA |
| 246 | Discovery | Healthy control | K0652 | 49 | F | NA | NA | NA | NA | NA | NA |
| 247 | Discovery | Healthy control | K0665 | 56 | F | NA | NA | NA | NA | NA | NA |
| 248 | Discovery | Healthy control | K0668 | 45 | F | NA | NA | NA | NA | NA | NA |
| 249 | Discovery | Healthy control | K0669 | 66 | F | NA | NA | NA | NA | NA | NA |
| 250 | Discovery | Healthy control | K0672 | 56 | F | NA | NA | NA | NA | NA | NA |
| 251 | Discovery | Healthy control | K0679 | 51 | F | NA | NA | NA | NA | NA | NA |
| 252 | Discovery | Healthy control | K0691 | 43 | F | NA | NA | NA | NA | NA | NA |
| 253 | Discovery | Healthy control | K0784 | 56 | F | NA | NA | NA | NA | NA | NA |
| 254 | Discovery | Healthy control | K1152 | 53 | F | NA | NA | NA | NA | NA | NA |
| 255 | Discovery | Healthy control | K1470 | 48 | F | NA | NA | NA | NA | NA | NA |
| 256 | Discovery | Healthy control | K1471 | 54 | F | NA | NA | NA | NA | NA | NA |
| 257 | Discovery | Healthy control | K1474 | 50 | F | NA | NA | NA | NA | NA | NA |
| 258 | Discovery | Healthy control | K1479 | 62 | F | NA | NA | NA | NA | NA | NA |
| 259 | Discovery | Healthy control | K1491 | 41 | F | NA | NA | NA | NA | NA | NA |
| 260 | Discovery | Healthy control | K1492 | 43 | F | NA | NA | NA | NA | NA | NA |
| 261 | Discovery | Healthy control | K1497 | 47 | F | NA | NA | NA | NA | NA | NA |
| 262 | Discovery | Healthy control | K1499 | 51 | F | NA | NA | NA | NA | NA | NA |
| 263 | Discovery | Healthy control | K1504 | 59 | F | NA | NA | NA | NA | NA | NA |
| 264 | Discovery | Healthy control | K1508 | 47 | F | NA | NA | NA | NA | NA | NA |
| 265 | Discovery | Healthy control | K1509 | 53 | F | NA | NA | NA | NA | NA | NA |
| 266 | Discovery | Healthy control | K1512 | 56 | F | NA | NA | NA | NA | NA | NA |
| 267 | Discovery | Healthy control | K1513 | 47 | F | NA | NA | NA | NA | NA | NA |
| 268 | Discovery | Healthy control | K1518 | 67 | F | NA | NA | NA | NA | NA | NA |
| 269 | Discovery | Healthy control | K1520 | 56 | F | NA | NA | NA | NA | NA | NA |
| 270 | Discovery | Healthy control | K1526 | 64 | F | NA | NA | NA | NA | NA | NA |
| 271 | Discovery | Healthy control | K1536 | 42 | F | NA | NA | NA | NA | NA | NA |
| 272 | Discovery | Healthy control | K1537 | 60 | F | NA | NA | NA | NA | NA | NA |
| 273 | Discovery | Healthy control | K1539 | 50 | F | NA | NA | NA | NA | NA | NA |
| 274 | Discovery | Healthy control | K1543 | 63 | F | NA | NA | NA | NA | NA | NA |
| 275 | Discovery | Healthy control | K1550 | 54 | F | NA | NA | NA | NA | NA | NA |
| 276 | Discovery | Healthy control | K1553 | 50 | F | NA | NA | NA | NA | NA | NA |
| 277 | Discovery | Healthy control | K1556 | 47 | F | NA | NA | NA | NA | NA | NA |
| 278 | Discovery | Healthy control | K1557 | 42 | F | NA | NA | NA | NA | NA | NA |
| 279 | Discovery | Healthy control | K1560 | 48 | F | NA | NA | NA | NA | NA | NA |
| 280 | Discovery | Healthy control | K1569 | 43 | F | NA | NA | NA | NA | NA | NA |
| 281 | Discovery | Healthy control | K1570 | 58 | F | NA | NA | NA | NA | NA | NA |
| 282 | Discovery | Healthy control | K1585 | 54 | F | NA | NA | NA | NA | NA | NA |
| 283 | Discovery | Healthy control | K1587 | 67 | F | NA | NA | NA | NA | NA | NA |
| 284 | Discovery | Healthy control | K1588 | 49 | F | NA | NA | NA | NA | NA | NA |
| 285 | Discovery | Healthy control | K1589 | 50 | F | NA | NA | NA | NA | NA | NA |
| 286 | Discovery | Healthy control | K1591 | 45 | F | NA | NA | NA | NA | NA | NA |
| 287 | Discovery | Healthy control | K1593 | 40 | F | NA | NA | NA | NA | NA | NA |
| 288 | Discovery | Healthy control | K1596 | 41 | F | NA | NA | NA | NA | NA | NA |
| 289 | Discovery | Healthy control | K1598 | 40 | F | NA | NA | NA | NA | NA | NA |
| 290 | Discovery | Healthy control | K1612 | 59 | F | NA | NA | NA | NA | NA | NA |
| 291 | Discovery | Healthy control | K1615 | 42 | F | NA | NA | NA | NA | NA | NA |
| 292 | Discovery | Healthy control | K1621 | 66 | F | NA | NA | NA | NA | NA | NA |
| 293 | Discovery | Healthy control | K1623 | 42 | F | NA | NA | NA | NA | NA | NA |
| 294 | Discovery | Healthy control | K1626 | 45 | F | NA | NA | NA | NA | NA | NA |
| 295 | Discovery | Healthy control | K1630 | 45 | F | NA | NA | NA | NA | NA | NA |
| 296 | Discovery | Healthy control | K1633 | 60 | F | NA | NA | NA | NA | NA | NA |
| 297 | Discovery | Healthy control | K1634 | 47 | F | NA | NA | NA | NA | NA | NA |
| 298 | Discovery | Healthy control | K1637 | 44 | F | NA | NA | NA | NA | NA | NA |
| 299 | Discovery | Healthy control | K1638 | 63 | F | NA | NA | NA | NA | NA | NA |
| 300 | Discovery | Healthy control | K1640 | 43 | F | NA | NA | NA | NA | NA | NA |
| 301 | Discovery | Healthy control | K1642 | 48 | F | NA | NA | NA | NA | NA | NA |
| 302 | Discovery | Healthy control | K1643 | 47 | F | NA | NA | NA | NA | NA | NA |
| 303 | Discovery | Healthy control | K1645 | 47 | F | NA | NA | NA | NA | NA | NA |
| 304 | Discovery | Healthy control | K1667 | 55 | F | NA | NA | NA | NA | NA | NA |
| 305 | Discovery | Healthy control | K1695 | 58 | F | NA | NA | NA | NA | NA | NA |
| 306 | Discovery | Healthy control | K1776 | 51 | F | NA | NA | NA | NA | NA | NA |
| 307 | Discovery | Healthy control | K1779 | 44 | F | NA | NA | NA | NA | NA | NA |
| 308 | Discovery | Healthy control | KAAA11 | 46 | F | NA | NA | NA | NA | NA | NA |
| 309 | Discovery | Healthy control | KAAA19 | 54 | F | NA | NA | NA | NA | NA | NA |
| 310 | Discovery | Healthy control | KAAA31 | 44 | F | NA | NA | NA | NA | NA | NA |
| 311 | Discovery | Healthy control | KAAA32 | 62 | F | NA | NA | NA | NA | NA | NA |
| 312 | Discovery | Healthy control | KAAA33 | 56 | F | NA | NA | NA | NA | NA | NA |
| 313 | Discovery | Healthy control | KAAA55 | 50 | F | NA | NA | NA | NA | NA | NA |
| 314 | Discovery | Healthy control | KAAA57 | 70 | F | NA | NA | NA | NA | NA | NA |
| 315 | Discovery | Healthy control | KAAA79 | 51 | F | NA | NA | NA | NA | NA | NA |
| 316 | Discovery | Healthy control | KAAA87 | 60 | F | NA | NA | NA | NA | NA | NA |
| 317 | Discovery | Healthy control | KAAA92 | 43 | F | NA | NA | NA | NA | NA | NA |
| 318 | Discovery | Healthy control | KAAB02 | 57 | F | NA | NA | NA | NA | NA | NA |
| 319 | Discovery | Healthy control | KAAB06 | 53 | F | NA | NA | NA | NA | NA | NA |
| 320 | Discovery | Healthy control | KAAB11 | 49 | F | NA | NA | NA | NA | NA | NA |
| 321 | Discovery | Healthy control | KAAB12 | 45 | F | NA | NA | NA | NA | NA | NA |
| 322 | Discovery | Healthy control | KAAB21 | 58 | F | NA | NA | NA | NA | NA | NA |
| 323 | Discovery | Healthy control | KAAB43 | 42 | F | NA | NA | NA | NA | NA | NA |
| 324 | Discovery | Healthy control | KAAB48 | 56 | F | NA | NA | NA | NA | NA | NA |
| 325 | Discovery | Healthy control | KAAB49 | 43 | F | NA | NA | NA | NA | NA | NA |
| 326 | Discovery | Healthy control | KAAB52 | 60 | F | NA | NA | NA | NA | NA | NA |
| 327 | Discovery | Healthy control | KAAB55 | 65 | F | NA | NA | NA | NA | NA | NA |
| 328 | Discovery | Healthy control | KAAB58 | 46 | F | NA | NA | NA | NA | NA | NA |
| 329 | Discovery | Healthy control | KAAB61 | 46 | F | NA | NA | NA | NA | NA | NA |
| 330 | Discovery | Healthy control | KAAB63 | 44 | F | NA | NA | NA | NA | NA | NA |
| 331 | Discovery | Healthy control | KAAB71 | 66 | F | NA | NA | NA | NA | NA | NA |
| 332 | Discovery | Healthy control | KAAC18 | 36 | F | NA | NA | NA | NA | NA | NA |
| 333 | Discovery | Healthy control | KAAD36 | 57 | F | NA | NA | NA | NA | NA | NA |
| 334 | Discovery | Healthy control | KAAD70 | 64 | F | NA | NA | NA | NA | NA | NA |
| 335 | Discovery | Healthy control | KAAD79 | 50 | F | NA | NA | NA | NA | NA | NA |
| 336 | Discovery | Healthy control | KAAE24 | 54 | F | NA | NA | NA | NA | NA | NA |
| 337 | Discovery | Healthy control | LBGS001R | 40 | F | NA | NA | NA | NA | NA | NA |
| 338 | Discovery | Healthy control | LBGS002R | 37 | F | NA | NA | NA | NA | NA | NA |
| 339 | Discovery | Healthy control | LBGS004R | 41 | F | NA | NA | NA | NA | NA | NA |
| 340 | Discovery | Healthy control | LBGS006 | 31 | F | NA | NA | NA | NA | NA | NA |
| 341 | Discovery | Healthy control | LBGS007R | 33 | F | NA | NA | NA | NA | NA | NA |
| 342 | Discovery | Healthy control | LBGS014 | 32 | F | NA | NA | NA | NA | NA | NA |
| 343 | Discovery | Healthy control | LBGS015 | 50 | F | NA | NA | NA | NA | NA | NA |
| 344 | Discovery | Healthy control | LBGS016 | 37 | F | NA | NA | NA | NA | NA | NA |
| 345 | Discovery | Healthy control | LBGS017 | 62 | F | NA | NA | NA | NA | NA | NA |
| 346 | Discovery | Healthy control | LBGS019 | 32 | F | NA | NA | NA | NA | NA | NA |
| 347 | Discovery | Healthy control | LBGS020R | 34 | F | NA | NA | NA | NA | NA | NA |
| 348 | Discovery | Healthy control | LBGS021 | 30 | F | NA | NA | NA | NA | NA | NA |
| 349 | Validation | Breast cancer | LBMB198 | 63 | F | IIA | Carcinoma | No | 1 | 25x20mm | Luminal B - HER2 |
| 350 | Validation | Breast cancer | LBMB205 | 38 | F | II | Carcinoma | Yes | NA | NA | Luminal B |
| 351 | Validation | Breast cancer | LBMB211 | 57 | F | II | Carcinoma | No | NA | NA | Luminal A |
| 352 | Validation | Breast cancer | LBMB219 | 39 | F | NA | Carcinoma | NA | NA | NA | Luminal B |
| 353 | Validation | Breast cancer | LBMB224 | 41 | F | NA | Carcinoma | NA | NA | NA | NA |
| 354 | Validation | Breast cancer | LBMB227 | 39 | F | NA | Carcinoma | NA | NA | NA | NA |
| 355 | Validation | Breast cancer | LBMB228 | 38 | F | NA | Carcinoma | NA | NA | NA | NA |
| 356 | Validation | Breast cancer | LBMB229 | 51 | F | NA | Carcinoma | NA | NA | NA | Luminal B |
| 357 | Validation | Breast cancer | LBMB236 | 47 | F | II | Carcinoma | Yes | 1 | 13x14mm | Luminal A |
| 358 | Validation | Breast cancer | LBMB237 | 49 | F | NA | Carcinoma | NA | NA | NA | NA |
| 359 | Validation | Breast cancer | LBMB238 | 38 | F | IA | Carcinoma | No | 1 | 11x11mm | Luminal A |
| 360 | Validation | Breast cancer | LBMB247 | 66 | F | NA | Carcinoma | NA | NA | NA | NA |
| 361 | Validation | Breast cancer | LBMB261 | 56 | F | NA | Carcinoma | NA | NA | NA | NA |
| 362 | Validation | Breast cancer | LBMB266 | 57 | F | NA | Carcinoma | NA | NA | NA | NA |
| 363 | Validation | Breast cancer | LBMB272 | 61 | F | I | Carcinoma | No | 1 | 11x12mm | NA |
| 364 | Validation | Breast cancer | LBMB287 | 70 | F | NA | Carcinoma | NA | NA | NA | NA |
| 365 | Validation | Breast cancer | LBMB299 | 64 | F | NA | Carcinoma | NA | NA | NA | NA |
| 366 | Validation | Breast cancer | LBMB300 | 43 | F | NA | Carcinoma | NA | NA | NA | NA |
| 367 | Validation | Breast cancer | MB003AR | 57 | F | II | Carcinoma | Yes | 1 | 62x30mm | HER2 |
| 368 | Validation | Breast cancer | MB013 | 63 | F | II | Carcinoma | No | 1 | 21x20mm | HER2 |
| 369 | Validation | Breast cancer | MB014AR | 52 | F | II | Carcinoma | No | 1 | 14x47mm | HER2 |
| 370 | Validation | Breast cancer | MB022 | 28 | F | II | Carcinoma | No | 1 | 7x10cm | Luminal B |
| 371 | Validation | Breast cancer | MB024 | 58 | F | II | Carcinoma | No | 1 | 17x14mm | Luminal B |
| 372 | Validation | Breast cancer | MB025 | 35 | F | II | Carcinoma | NA | 1 | 22x10mm | NA |
| 373 | Validation | Breast cancer | MB027AR | 58 | F | II | Carcinoma | Yes | 1 | 40mm | HER2 |
| 374 | Validation | Breast cancer | MB029A | 32 | F | I | Carcinoma | No | 1 | 16x8mm | Luminal B - HER2 |
| 375 | Validation | Breast cancer | MB030A | 48 | F | II | Carcinoma | Yes | 1 | 12x21mm | Luminal B |
| 376 | Validation | Breast cancer | MB033 | 51 | F | II | Carcinoma | No | 1 | 32x11mm | HER2 |
| 377 | Validation | Breast cancer | MB034A | 55 | F | II | Carcinoma | No | 1 | 28x12mm | HER2 |
| 378 | Validation | Breast cancer | MB035 | 48 | F | II | Carcinoma | No | 2 | 50x29mm, 22x14mm | Luminal B |
| 379 | Validation | Breast cancer | MB039A | 52 | F | I | Carcinoma | No | 1 | 12x6mm | Luminal B |
| 380 | Validation | Breast cancer | MB042A | 69 | F | II | Carcinoma | No | 1 | 20x22mm | Luminal A |
| 381 | Validation | Breast cancer | MB045A | 78 | F | I | Carcinoma | No | 1 | 14x13mm | Luminal A |
| 382 | Validation | Breast cancer | MB051AR | 60 | F | II | Carcinoma | No | 1 | 23x13mm | HER2 |
| 383 | Validation | Breast cancer | MB052 | 51 | F | III | Carcinoma | Yes | 2 | 42x25mm, 10x9mm | Luminal B |
| 384 | Validation | Breast cancer | MB053A | 45 | F | II | Carcinoma | No | 1 | 60x45mm | Luminal B |
| 385 | Validation | Breast cancer | MB057A | 57 | F | II | Carcinoma | Yes | 1 | 18x15mm | Luminal B - HER2 |
| 386 | Validation | Breast cancer | MB069A | 57 | F | II | Carcinoma | No | 1 | 24x23mm | Luminal B |
| 387 | Validation | Breast cancer | MB071AR | 66 | F | II | Carcinoma | No | 1 | 10x20mm | Luminal A |
| 388 | Validation | Breast cancer | MB073 | 40 | F | II | Carcinoma | Yes | 1 | 15x25mm | Luminal B |
| 389 | Validation | Breast cancer | MB074A | 45 | F | I | Carcinoma | No | 1 | 12x25mm | Luminal B |
| 390 | Validation | Breast cancer | MB076A | 51 | F | II | Carcinoma | No | 1 | 35mm | Luminal B |
| 391 | Validation | Breast cancer | MB082A | 58 | F | III | Carcinoma | Yes | 1 | 26x16mm | Luminal B |
| 392 | Validation | Breast cancer | MB083AR | 57 | F | III | Carcinoma | Yes | 1 | 40x30x30mm | HER2 |
| 393 | Validation | Breast cancer | MB092 | 60 | F | III | Carcinoma | Yes | 1 | 26mm | HER2 |
| 394 | Validation | Breast cancer | MB097 | 31 | F | III | Carcinoma | NA | 1 | 19x14mm | NA |
| 395 | Validation | Breast cancer | MB098 | 56 | F | II | Carcinoma | No | 1 | 20x12mm | Luminal B |
| 396 | Validation | Breast cancer | MB101 | 55 | F | II | Carcinoma | Yes | 1 | 22x24mm | HER2 |
| 397 | Validation | Breast cancer | MB106A | 51 | F | NA | Carcinoma | NA | 1 | 4cm | NA |
| 398 | Validation | Breast cancer | MB107 | 41 | F | IA | Carcinoma | No | 1 | 15x20mm | Luminal B |
| 399 | Validation | Breast cancer | MB133 | 62 | F | II | Carcinoma | Yes | 1 | 3cm | Luminal B - HER2 |
| 400 | Validation | Breast cancer | MB134 | 32 | F | IIIA | Carcinoma | Yes | 1 | 4x5cm | Luminal B |
| 401 | Validation | Breast cancer | MB149 | 60 | F | NA | Carcinoma | NA | 1 | 23x23x29mm | TNBC |
| 402 | Validation | Breast cancer | MB205 | 46 | F | II | Carcinoma | No | 1 | 25mm | Luminal A |
| 403 | Validation | Breast cancer | MB207 | 67 | F | I | Carcinoma | Yes | 1 | 16x15mm | Luminal A |
| 404 | Validation | Breast cancer | MB211A | 56 | F | III | Carcinoma | Yes | 1 | 47x38mm | HER2 |
| 405 | Validation | Breast cancer | MB213 | 35 | F | I | Carcinoma | No | 1 | 19x10mm | Luminal B |
| 406 | Validation | Breast cancer | MB214 | 32 | F | II | Carcinoma | No | 1 | 10x23mm | Luminal B - HER2 |
| 407 | Validation | Breast cancer | NB01 | 48 | F | III | Carcinoma | No | 1 | 7x5cm | HER2 |
| 408 | Validation | Breast cancer | NB024 | 52 | F | I | Carcinoma | No | 1 | 15x10mm | Luminal B |
| 409 | Validation | Breast cancer | NB029 | 51 | F | II | Carcinoma | NA | 1 | 26x20mm | Luminal B - HER2 |
| 410 | Validation | Breast cancer | NB03 | 39 | F | III | Carcinoma | Yes | 1 | 36mm | Luminal B |
| 411 | Validation | Breast cancer | NB033 | 47 | F | II | Carcinoma | No | 1 | 25mm | Luminal B |
| 412 | Validation | Breast cancer | NB034 | 50 | F | II | Carcinoma | No | 1 | 27x15mm | Luminal B |
| 413 | Validation | Breast cancer | NB036 | 64 | F | III | Carcinoma | Yes | 1 | 17x13,6mm | Luminal B |
| 414 | Validation | Breast cancer | NB040 | 44 | F | III | Carcinoma | Yes | 1 | 4cm | TNBC |
| 415 | Validation | Breast cancer | NB05R2 | 71 | F | I | Carcinoma | No | 1 | 1cm | HER2 |
| 416 | Validation | Breast cancer | NB06R2 | 49 | F | III | Carcinoma | Yes | 1 | 32x18mm | HER2 |
| 417 | Validation | Breast cancer | NB07R2 | 44 | F | II | Carcinoma | No | 1 | 21x13mm | Luminal B |
| 418 | Validation | Breast cancer | NB08 | 68 | F | I | Carcinoma | No | 1 | 17x9mm | HER2 |
| 419 | Validation | Breast cancer | NB21 | 53 | F | III | Carcinoma | Yes | 1 | 28x8mm | HER2 |
| 420 | Validation | Breast cancer | NB47 | 48 | F | III | Carcinoma | Yes | 1 | 53mm | Luminal B - HER2 |
| 421 | Validation | Healthy control | K0004 | 46 | F | NA | NA | NA | NA | NA | NA |
| 422 | Validation | Healthy control | K0006 | 61 | F | NA | NA | NA | NA | NA | NA |
| 423 | Validation | Healthy control | K0009 | 66 | F | NA | NA | NA | NA | NA | NA |
| 424 | Validation | Healthy control | K0014 | 42 | F | NA | NA | NA | NA | NA | NA |
| 425 | Validation | Healthy control | K0020 | 40 | F | NA | NA | NA | NA | NA | NA |
| 426 | Validation | Healthy control | K0052 | 50 | F | NA | NA | NA | NA | NA | NA |
| 427 | Validation | Healthy control | K0059 | 62 | F | NA | NA | NA | NA | NA | NA |
| 428 | Validation | Healthy control | K0071 | 40 | F | NA | NA | NA | NA | NA | NA |
| 429 | Validation | Healthy control | K0107 | 41 | F | NA | NA | NA | NA | NA | NA |
| 430 | Validation | Healthy control | K0111 | 49 | F | NA | NA | NA | NA | NA | NA |
| 431 | Validation | Healthy control | K0114 | 49 | F | NA | NA | NA | NA | NA | NA |
| 432 | Validation | Healthy control | K0119 | 46 | F | NA | NA | NA | NA | NA | NA |
| 433 | Validation | Healthy control | K0121 | 46 | F | NA | NA | NA | NA | NA | NA |
| 434 | Validation | Healthy control | K0135 | 52 | F | NA | NA | NA | NA | NA | NA |
| 435 | Validation | Healthy control | K0161 | 42 | F | NA | NA | NA | NA | NA | NA |
| 436 | Validation | Healthy control | K0162 | 51 | F | NA | NA | NA | NA | NA | NA |
| 437 | Validation | Healthy control | K0173 | 55 | F | NA | NA | NA | NA | NA | NA |
| 438 | Validation | Healthy control | K0177 | 50 | F | NA | NA | NA | NA | NA | NA |
| 439 | Validation | Healthy control | K0189 | 54 | F | NA | NA | NA | NA | NA | NA |
| 440 | Validation | Healthy control | K0191 | 42 | F | NA | NA | NA | NA | NA | NA |
| 441 | Validation | Healthy control | K0200 | 40 | F | NA | NA | NA | NA | NA | NA |
| 442 | Validation | Healthy control | K0228 | 62 | F | NA | NA | NA | NA | NA | NA |
| 443 | Validation | Healthy control | K0270 | 57 | F | NA | NA | NA | NA | NA | NA |
| 444 | Validation | Healthy control | K0282 | 47 | F | NA | NA | NA | NA | NA | NA |
| 445 | Validation | Healthy control | K0287 | 62 | F | NA | NA | NA | NA | NA | NA |
| 446 | Validation | Healthy control | K0293 | 46 | F | NA | NA | NA | NA | NA | NA |
| 447 | Validation | Healthy control | K0296 | 41 | F | NA | NA | NA | NA | NA | NA |
| 448 | Validation | Healthy control | K0304 | 46 | F | NA | NA | NA | NA | NA | NA |
| 449 | Validation | Healthy control | K0308 | 64 | F | NA | NA | NA | NA | NA | NA |
| 450 | Validation | Healthy control | K0313 | 66 | F | NA | NA | NA | NA | NA | NA |
| 451 | Validation | Healthy control | K0423 | 52 | F | NA | NA | NA | NA | NA | NA |
| 452 | Validation | Healthy control | K0428 | 51 | F | NA | NA | NA | NA | NA | NA |
| 453 | Validation | Healthy control | K0433 | 53 | F | NA | NA | NA | NA | NA | NA |
| 454 | Validation | Healthy control | K0434 | 68 | F | NA | NA | NA | NA | NA | NA |
| 455 | Validation | Healthy control | K0466 | 50 | F | NA | NA | NA | NA | NA | NA |
| 456 | Validation | Healthy control | K0579 | 49 | F | NA | NA | NA | NA | NA | NA |
| 457 | Validation | Healthy control | K0585 | 43 | F | NA | NA | NA | NA | NA | NA |
| 458 | Validation | Healthy control | K0603 | 48 | F | NA | NA | NA | NA | NA | NA |
| 459 | Validation | Healthy control | K0633 | 55 | F | NA | NA | NA | NA | NA | NA |
| 460 | Validation | Healthy control | K0670 | 56 | F | NA | NA | NA | NA | NA | NA |
| 461 | Validation | Healthy control | K0683 | 44 | F | NA | NA | NA | NA | NA | NA |
| 462 | Validation | Healthy control | K0779 | 56 | F | NA | NA | NA | NA | NA | NA |
| 463 | Validation | Healthy control | K1464 | 40 | F | NA | NA | NA | NA | NA | NA |
| 464 | Validation | Healthy control | K1478 | 48 | F | NA | NA | NA | NA | NA | NA |
| 465 | Validation | Healthy control | K1483 | 50 | F | NA | NA | NA | NA | NA | NA |
| 466 | Validation | Healthy control | K1489 | 53 | F | NA | NA | NA | NA | NA | NA |
| 467 | Validation | Healthy control | K1500 | 54 | F | NA | NA | NA | NA | NA | NA |
| 468 | Validation | Healthy control | K1505 | 59 | F | NA | NA | NA | NA | NA | NA |
| 469 | Validation | Healthy control | K1507 | 58 | F | NA | NA | NA | NA | NA | NA |
| 470 | Validation | Healthy control | K1516 | 62 | F | NA | NA | NA | NA | NA | NA |
| 471 | Validation | Healthy control | K1519 | 65 | F | NA | NA | NA | NA | NA | NA |
| 472 | Validation | Healthy control | K1523 | 69 | F | NA | NA | NA | NA | NA | NA |
| 473 | Validation | Healthy control | K1530 | 46 | F | NA | NA | NA | NA | NA | NA |
| 474 | Validation | Healthy control | K1533 | 50 | F | NA | NA | NA | NA | NA | NA |
| 475 | Validation | Healthy control | K1568 | 52 | F | NA | NA | NA | NA | NA | NA |
| 476 | Validation | Healthy control | K1614 | 57 | F | NA | NA | NA | NA | NA | NA |
| 477 | Validation | Healthy control | K1629 | 42 | F | NA | NA | NA | NA | NA | NA |
| 478 | Validation | Healthy control | K1641 | 45 | F | NA | NA | NA | NA | NA | NA |
| 478 | Validation | Healthy control | K1668 | 55 | F | NA | NA | NA | NA | NA | NA |
| 480 | Validation | Healthy control | K1693 | 52 | F | NA | NA | NA | NA | NA | NA |
| 481 | Validation | Healthy control | K1697 | 61 | F | NA | NA | NA | NA | NA | NA |
| 482 | Validation | Healthy control | K1778 | 50 | F | NA | NA | NA | NA | NA | NA |
| 483 | Validation | Healthy control | KAAA18 | 59 | F | NA | NA | NA | NA | NA | NA |
| 484 | Validation | Healthy control | KAAA20 | 44 | F | NA | NA | NA | NA | NA | NA |
| 485 | Validation | Healthy control | KAAA44 | 42 | F | NA | NA | NA | NA | NA | NA |
| 486 | Validation | Healthy control | KAAA51 | 54 | F | NA | NA | NA | NA | NA | NA |
| 487 | Validation | Healthy control | KAAA73 | 52 | F | NA | NA | NA | NA | NA | NA |
| 488 | Validation | Healthy control | KAAA81 | 58 | F | NA | NA | NA | NA | NA | NA |
| 489 | Validation | Healthy control | KAAA83 | 53 | F | NA | NA | NA | NA | NA | NA |
| 490 | Validation | Healthy control | KAAA97 | 60 | F | NA | NA | NA | NA | NA | NA |
| 491 | Validation | Healthy control | KAAB46 | 55 | F | NA | NA | NA | NA | NA | NA |
| 492 | Validation | Healthy control | KAAB76 | 42 | F | NA | NA | NA | NA | NA | NA |
| 493 | Validation | Healthy control | KAAC99 | 54 | F | NA | NA | NA | NA | NA | NA |
| 494 | Validation | Healthy control | KAAD65 | 54 | F | NA | NA | NA | NA | NA | NA |
| 495 | Validation | Healthy control | KAAD76 | 45 | F | NA | NA | NA | NA | NA | NA |
| 496 | Validation | Healthy control | KAAD81 | 59 | F | NA | NA | NA | NA | NA | NA |
| 497 | Validation | Healthy control | KAAE40 | 68 | F | NA | NA | NA | NA | NA | NA |
| 498 | Validation | Healthy control | LBGS005R | 32 | F | NA | NA | NA | NA | NA | NA |
| 499 | Validation | Healthy control | LBGS008 | 47 | F | NA | NA | NA | NA | NA | NA |
| 500 | Validation | Healthy control | LBGS009 | 34 | F | NA | NA | NA | NA | NA | NA |
| 501 | Validation | Healthy control | LBGS010R | 36 | F | NA | NA | NA | NA | NA | NA |
| 502 | Validation | Healthy control | LBGS013 | 39 | F | NA | NA | NA | NA | NA | NA |
| 503 | Validation | Healthy control | LBGS023R | 39 | F | NA | NA | NA | NA | NA | NA |
| 504 | Validation | Healthy control | LBGS027 | 38 | F | NA | NA | NA | NA | NA | NA |
| 505 | Validation | Healthy control | LBGS045R | 32 | F | NA | NA | NA | NA | NA | NA |
| 506 | Validation | Healthy control | LBGS047R | 31 | F | NA | NA | NA | NA | NA | NA |
| 507 | Validation | Healthy control | LBGS051R | 37 | F | NA | NA | NA | NA | NA | NA |
| 508 | Validation | Healthy control | LBGS052R | 37 | F | NA | NA | NA | NA | NA | NA |
| 509 | Validation | Healthy control | LBGS054 | 33 | F | NA | NA | NA | NA | NA | NA |
| 510 | Validation | Healthy control | LBGS055 | 36 | F | NA | NA | NA | NA | NA | NA |
| 511 | Validation | Healthy control | LBGS062 | 45 | F | NA | NA | NA | NA | NA | NA |
| 512 | Validation | Healthy control | LBGS067 | 50 | F | NA | NA | NA | NA | NA | NA |
| 513 | Validation | Healthy control | LBGS075 | 41 | F | NA | NA | NA | NA | NA | NA |
| 514 | Validation | Healthy control | LBGS076 | 77 | F | NA | NA | NA | NA | NA | NA |
| 515 | Validation | Healthy control | LBGS080R | 59 | F | NA | NA | NA | NA | NA | NA |
| 516 | Validation | Healthy control | LBGS103R | 64 | F | NA | NA | NA | NA | NA | NA |
| 517 | Validation | Healthy control | LBGS111 | 58 | F | NA | NA | NA | NA | NA | NA |

| **Table S2A.** Summary of HCC patients' clinical features | | | |
| --- | --- | --- | --- |
| **Criteria** | | **HCC  (N = 87)** | |
|  |  | **N** | **Percentage** |
| Gender | Female | 20 | 23.0% |
|  | Male | 67 | 77.0% |
| Age | Median | 58 |  |
|  | Min | 27 |  |
|  | Max | 86 |  |
| Stage | I | 6 | 6.9% |
|  | II | 21 | 24.1% |
|  | III | 0 | 0.0% |
|  | IV | 1 | 1.1% |
|  | NA | 59 | 67.8% |

| **Table S2B.** Clinical characteristics of HCC patients | | | | | |
| --- | --- | --- | --- | --- | --- |
| **No** | **SampleID** | **Age** | **Gender** | **Tumor Stage** | **Tumor type** |
| 1 | LBH001 | 40 | M | II | HCC |
| 2 | LBH002 | 86 | M | I | HCC |
| 3 | LBH003 | 37 | M | NA | HCC |
| 4 | LBH004 | 35 | M | I | HCC |
| 5 | LBH005 | 56 | M | NA | HCC |
| 6 | LBH006 | 50 | M | II | HCC |
| 7 | LBH007 | 57 | F | NA | HCC |
| 8 | LBH009 | 51 | M | NA | HCC |
| 9 | LBH010 | 68 | M | NA | HCC |
| 10 | LBH011 | 49 | M | I | HCC |
| 11 | LBH012 | 55 | M | NA | HCC |
| 12 | LBH013 | 76 | M | NA | HCC |
| 13 | LBH019 | 34 | M | II | HCC |
| 14 | LBH024 | 70 | F | NA | HCC |
| 15 | LBH025 | 63 | M | II | HCC |
| 16 | LBH026 | 62 | M | II | HCC |
| 17 | LBH027 | 27 | M | II | HCC |
| 18 | LBH028 | 51 | M | II | HCC |
| 19 | LBH030 | 66 | M | II | HCC |
| 20 | LBH031 | 69 | M | II | HCC |
| 21 | LBH032 | 55 | M | II | HCC |
| 22 | LBH033 | 49 | M | II | HCC |
| 23 | LBH034 | 67 | F | II | HCC |
| 24 | LBH035 | 58 | M | II | HCC |
| 25 | LBH036 | 57 | M | II | HCC |
| 26 | LBH037 | 59 | M | I | HCC |
| 27 | LBH038 | 63 | F | II | HCC |
| 28 | LBH039 | 73 | M | I | HCC |
| 29 | LBH040 | 58 | M | II | HCC |
| 30 | LBH041 | 52 | M | II | HCC |
| 31 | LBH042 | 63 | F | IV | HCC |
| 32 | LBH045 | 35 | M | II | HCC |
| 33 | LBH047 | 63 | M | NA | HCC |
| 34 | LBH048 | 46 | M | NA | HCC |
| 35 | LBH049 | 52 | M | II | HCC |
| 36 | LBH050 | 58 | M | II | HCC |
| 37 | LBH051 | 70 | F | NA | HCC |
| 38 | LBH052 | 54 | M | II | HCC |
| 39 | LBH054 | 57 | M | I | HCC |
| 40 | LBH055 | 35 | M | NA | HCC |
| 41 | LBH056 | 60 | F | NA | HCC |
| 42 | LBM005 | 57 | M | NA | HCC |
| 43 | LBM006 | 72 | M | NA | HCC |
| 44 | LBM010 | 46 | M | NA | HCC |
| 45 | LBM016 | 32 | M | NA | HCC |
| 46 | LBM018 | 62 | M | NA | HCC |
| 47 | LBM022 | 68 | M | NA | HCC |
| 48 | LBM023 | 65 | M | NA | HCC |
| 49 | LBM025 | 68 | F | NA | HCC |
| 50 | LBM028 | 76 | F | NA | HCC |
| 51 | LBM031 | 51 | M | NA | HCC |
| 52 | LBM033 | 55 | M | NA | HCC |
| 53 | LBM036 | 61 | F | NA | HCC |
| 54 | LBM037 | 47 | M | NA | HCC |
| 55 | LBM038 | 72 | M | NA | HCC |
| 56 | LBM040 | 59 | F | NA | HCC |
| 57 | LBM043 | 64 | M | NA | HCC |
| 58 | LBM047 | 71 | M | NA | HCC |
| 59 | LBM049 | 80 | F | NA | HCC |
| 60 | LBM052 | 56 | M | NA | HCC |
| 61 | LBM053 | 68 | M | NA | HCC |
| 62 | LBM054 | 54 | M | NA | HCC |
| 63 | LBM056 | 63 | F | NA | HCC |
| 64 | LBM059 | 52 | M | NA | HCC |
| 65 | LBM060 | 45 | F | NA | HCC |
| 66 | LBM063 | 81 | M | NA | HCC |
| 67 | LBM065 | 65 | M | NA | HCC |
| 68 | LBM070 | 70 | M | NA | HCC |
| 69 | LBM074 | 58 | M | NA | HCC |
| 70 | LBM076 | 63 | M | NA | HCC |
| 71 | LBM078 | 50 | M | NA | HCC |
| 72 | LBM083 | 54 | M | NA | HCC |
| 73 | LBM096 | 85 | M | NA | HCC |
| 74 | LBM111 | 70 | F | NA | HCC |
| 75 | LBM113 | 41 | M | NA | HCC |
| 76 | LBM124 | 60 | M | NA | HCC |
| 77 | LBM137 | 68 | M | NA | HCC |
| 78 | LBM145 | 49 | M | NA | HCC |
| 79 | LBM147 | 75 | F | NA | HCC |
| 80 | LBM168 | 64 | F | NA | HCC |
| 81 | LBM172 | 62 | F | NA | HCC |
| 82 | LBM187 | 58 | M | NA | HCC |
| 83 | LBM189 | 60 | M | NA | HCC |
| 84 | LBM194 | 43 | M | NA | HCC |
| 85 | LBM196 | 78 | F | NA | HCC |
| 86 | LBM204 | 54 | M | NA | HCC |
| 87 | LBM213 | 56 | F | NA | HCC |

**Table S3.** List of 16 potential DMRs for differentiating breast cancer from healthy individuals from literature search

| **region** | **chrom** | **start** | **end** | **Gene** | **Hyper / Hypomethylation** | **Detection method** | **Reference** | **p-value** | **log2FC** |
| --- | --- | --- | --- | --- | --- | --- | --- | --- | --- |
| TMD_1 | chr1 | 5E+07 | 5E+07 | DMRTA2 (1) | Hypomethylation | Illumina ChIP-Seq DNA | (1) | 0.2614 | 0.038682 |
| TMD_2 | chr1 | 5E+07 | 5E+07 | DMRTA2 (2) | Hypomethylation | Illumina ChIP-Seq DNA | (1) | 0.327 | 0.069035 |
| TMD_3 | chr1 | 5E+07 | 5E+07 | DMRTA2 (3) | Hypomethylation | Illumina ChIP-Seq DNA | (1) | 0.9409 | -0.24295 |
| TMD_4 | chr11 | 3.2E+07 | 3.2E+07 | PAX6 | Hypermethylation | MS–MLPA | (2) | 0.9024 | 0.037047 |
| TMD_5 | chr14 | 5.7E+07 | 5.7E+07 | **OTX2 (1)** | Hypermethylation | 450K array (TCGA dataset) | (3) | **0.0437** | 0.195001 |
| TMD_6 | chr14 | 5.7E+07 | 5.7E+07 | **OTX2 (2)** | Hypermethylation | 450K array (TCGA dataset) | (3) | **0.0383** | 0.244901 |
| TMD_7 | chr18 | 7.9E+07 | 7.9E+07 | SALL3 | Hypermethylation | 450K array (TCGA dataset) | (4) | 0.8561 | 0.000439 |
| TMD_8 | chr2 | 8.6E+07 | 8.6E+07 | ST3GAL5 | Hypermethylation | Multiple dataset | (5) | 0.0595 | -0.00774 |
| TMD_9 | chr3 | 5E+07 | 5E+07 | **RASSF1A** | Hypermethylation | MSP | (6) | **0.0042** | 0.622718 |
| TMD_10 | chr5 | 1.1E+08 | 1.1E+08 | **APC** | Hypermethylation | MSP | (7) | **0.0337** | -0.10839 |
| TMD_11 | chr6 | 1E+07 | 1E+07 | TFAP2A (1) | Hypermethylation | MSP | (8) | 0.2533 | 0.039815 |
| TMD_12 | chr6 | 1E+07 | 1E+07 | TFAP2A (2) | Hypermethylation | MSP | (8) | 0.3766 | 0.252508 |
| TMD_13 | chr7 | 1.5E+08 | 1.5E+08 | KCNH2 | Hypermethylation | MSE-PCR | (9) | 0.5098 | 0.013869 |
| TMD_14 | chr8 | 5.4E+07 | 5.4E+07 | SOX17 (1) | Hypermethylation | MSP | (10) | 0.3977 | 0.316574 |
| TMD_15 | chr8 | 5.4E+07 | 5.4E+07 | SOX17 (2) | Hypermethylation | MSP | (10) | 0.0028 | 0.23255 |
| TMD_16 | chr8 | 9.2E+07 | 9.2E+07 | RUNX1T1 | Hypermethylation | Illumina ChIP-Seq DNA | (1) | 0.2908 | 0.001127 |
| TMD_17 | chr9 | 2.2E+07 | 2.2E+07 | P16 (CDKN2A) | Hypermethylation | Quantitative Region-Specific DNA Methylation Analysis by the EpiTYPER™ | (11) | 0.7219 | -0.09182 |

MSP: Methylation-specific polymerase chain reaction

MSE: Methylation specific electrophoresis

MS-MLPA: Methylation-Specific Multiplex Ligation-Dependent Probe Amplification

Regions confirmed to be differentially methylated in our study are in bold

**Table S4.** Overview of ctDNA based assays for breast cancer detection in recent publications

| **Assay** | **Pub. Year** | **Methods** | **Features** | **ML technique** | **Total samples** | **Performance on breast cancer** | **Depth of sequencing** | **Ref.** |
| --- | --- | --- | --- | --- | --- | --- | --- | --- |
| CancerSEEK | 2018 | Amplicon sequencing Protein blood test | Mutation  Protein biomarker | Logistic regression | 1817 | Sensitivity: 33% Specificity > 99% | NA | (12) |
| DELFI | 2019 | Whole-genome sequencing | Fragment profile | Gradient tree boosting model | 481 | Sensitivity: 57% Specificity: 98% | Genome-wide: 1–2X | (13) |
| Galleri | 2020 | Whole-genome bisulfite sequencing  Targeted methylation sequencing | Methylation patterns | Ensemble of logistic regression | 5309 | Sensitivity: 30.5%  Specificity: 99.5% | Target: 139X  Genome-wide: 30X | (14) |
| DEEPGENTM | 2021 | Target sequencing | Mutation | Random forest | 675 | Sensitivity: 16%  Specificity: 99% | Target: 200,000X | (15) |

**References**

1. Stirzaker C, Zotenko E, Song JZ, Qu W, Nair SS, Locke WJ, Stone A, Armstong NJ, Robinson MD, Dobrovic A, Avery-Kiejda KA, Peters KM, French JD, Stein S, Korbie DJ, Trau M, Forbes JF, Scott RJ, Brown MA, Francis GD, Clark SJ. Methylome sequencing in triple-negative breast cancer reveals distinct methylation clusters with prognostic value. Nat Commun. 2015;6:5899.

2. Moelans CB, Verschuur-Maes AHJ, van Diest PJ. Frequent promoter hypermethylation of BRCA2, CDH13, MSH6, PAX5, PAX6 and WT1 in ductal carcinoma in situ and invasive breast cancer. The Journal of Pathology. 2011;225(2):222-31.

3. Guo Y, Mao X, Qiao Z, Chen B, Jin F. A Novel Promoter CpG-Based Signature for Long-Term Survival Prediction of Breast Cancer Patients. Frontiers in Oncology. 2020;10.

4. Zhang C, Zhao H, Li J, Liu H, Wang F, Wei Y, Su J, Zhang D, Liu T, Zhang Y. The Identification of Specific Methylation Patterns across Different Cancers. PLOS ONE. 2015;10(3):e0120361.

5. Ouyang S, Liu JH, Ni Z, Ding GF, Wang QZ. Downregulation of ST3GAL5 is associated with muscle invasion, high grade and a poor prognosis in patients with bladder cancer. Oncol Lett. 2020;20(1):828-40.

6. Dammann R, Yang G, Pfeifer GP. Hypermethylation of the CpG Island of Ras Association Domain Family 1A (RASSF1A), a Putative Tumor Suppressor Gene from the 3p21.3 Locus, Occurs in a Large Percentage of Human Breast Cancers1. Cancer Research. 2001;61(7):3105-9.

7. Jin Z, Tamura G, Tsuchiya T, Sakata K, Kashiwaba M, Osakabe M, Motoyama T. Adenomatous polyposis coli (APC) gene promoter hypermethylation in primary breast cancers. British Journal of Cancer. 2001;85(1):69-73.

8. Douglas DB, Akiyama Y, Carraway H, Belinsky SA, Esteller M, Gabrielson E, Weitzman S, Williams T, Herman JG, Baylin SB. Hypermethylation of a Small CpGuanine-Rich Region Correlates with Loss of Activator Protein-2α Expression during Progression of Breast Cancer. Cancer Research. 2004;64(5):1611-20.

9. Kuznetsova EB, Kekeeva TV, Larin SS, Zemlyakova VV, Babenko OV, Nemtsova MV, Zaletayev DV, Strelnikov VV. Novel markers of gene methylation and expression in breast cancer. Molecular Biology. 2007;41(4):562-70.

10. Chimonidou M, Strati A, Malamos N, Georgoulias V, Lianidou ES. SOX17 Promoter Methylation in Circulating Tumor Cells and Matched Cell-Free DNA Isolated from Plasma of Patients with Breast Cancer. Clinical Chemistry. 2013;59(1):270-9.

11. Radpour R, Barekati Z, Kohler C, Lv Q, Bürki N, Diesch C, Bitzer J, Zheng H, Schmid S, Zhong XY. Hypermethylation of Tumor Suppressor Genes Involved in Critical Regulatory Pathways for Developing a Blood-Based Test in Breast Cancer. PLOS ONE. 2011;6(1):e16080.

12. Cohen JD, Li L, Wang Y, Thoburn C, Afsari B, Danilova L, Douville C, Javed AA, Wong F, Mattox A, Hruban RH, Wolfgang CL, Goggins MG, Dal Molin M, Wang TL, Roden R, Klein AP, Ptak J, Dobbyn L, Schaefer J, Silliman N, Popoli M, Vogelstein JT, Browne JD, Schoen RE, Brand RE, Tie J, Gibbs P, Wong HL, Mansfield AS, Jen J, Hanash SM, Falconi M, Allen PJ, Zhou S, Bettegowda C, Diaz LA, Jr., Tomasetti C, Kinzler KW, Vogelstein B, Lennon AM, Papadopoulos N. Detection and localization of surgically resectable cancers with a multi-analyte blood test. Science. 2018;359(6378):926-30.

13. Cristiano S, Leal A, Phallen J, Fiksel J, Adleff V, Bruhm DC, Jensen SØ, Medina JE, Hruban C, White JR, Palsgrove DN, Niknafs N, Anagnostou V, Forde P, Naidoo J, Marrone K, Brahmer J, Woodward BD, Husain H, van Rooijen KL, Ørntoft M-BW, Madsen AH, van de Velde CJH, Verheij M, Cats A, Punt CJA, Vink GR, van Grieken NCT, Koopman M, Fijneman RJA, Johansen JS, Nielsen HJ, Meijer GA, Andersen CL, Scharpf RB, Velculescu VE. Genome-wide cell-free DNA fragmentation in patients with cancer. Nature. 2019;570(7761):385-9.

14. Klein EA, Richards D, Cohn A, Tummala M, Lapham R, Cosgrove D, Chung G, Clement J, Gao J, Hunkapiller N, Jamshidi A, Kurtzman KN, Seiden MV, Swanton C, Liu MC. Clinical validation of a targeted methylation-based multi-cancer early detection test using an independent validation set. Ann Oncol. 2021;32(9):1167-77.

15. Ris F, Hellan M, Douissard J, Nieva JJ, Triponez F, Woo Y, Geller D, Buchs NC, Buehler L, Moenig S, Iselin CE, Karenovics W, Petignat P, Lam GT, Undurraga Malinervo M, Tuttle R, Ouellette J, Bose D, Ismail N, Toso C. Blood-Based Multi-Cancer Detection Using a Novel Variant Calling Assay (DEEPGEN(TM)): Early Clinical Results. Cancers (Basel). 2021;13(16).
